# Comparative Phylogeography of a Coevolved Community: Concerted Population Expansions in Joshua Trees and Four Yucca Moths

**DOI:** 10.1371/journal.pone.0025628

**Published:** 2011-10-18

**Authors:** Christopher Irwin Smith, Shantel Tank, William Godsoe, Jim Levenick, Eva Strand, Todd Esque, Olle Pellmyr

**Affiliations:** 1 Department of Biology, Willamette University, Salem, Oregon, United States of America; 2 Department of Biological Sciences, University of Idaho, Moscow, Idaho, United States of America; 3 Department of Computer Science, Willamette University, Salem, Oregon, United States of America; 4 Department of Forest Ecology and Biogeosciences, University of Idaho, Moscow, Idaho, United States of America; 5 Western Ecological Research Centre, US Geological Survey, Henderson, Nevada, United States of America; Montreal Botanical Garden, Canada

## Abstract

Comparative phylogeographic studies have had mixed success in identifying common phylogeographic patterns among co-distributed organisms. Whereas some have found broadly similar patterns across a diverse array of taxa, others have found that the histories of different species are more idiosyncratic than congruent. The variation in the results of comparative phylogeographic studies could indicate that the extent to which sympatrically-distributed organisms share common biogeographic histories varies depending on the strength and specificity of ecological interactions between them. To test this hypothesis, we examined demographic and phylogeographic patterns in a highly specialized, coevolved community – Joshua trees (*Yucca brevifolia*) and their associated yucca moths. This tightly-integrated, mutually interdependent community is known to have experienced significant range changes at the end of the last glacial period, so there is a strong *a priori* expectation that these organisms will show common signatures of demographic and distributional changes over time. Using a database of >5000 GPS records for Joshua trees, and multi-locus DNA sequence data from the Joshua tree and four species of yucca moth, we combined paleaodistribution modeling with coalescent-based analyses of demographic and phylgeographic history. We extensively evaluated the power of our methods to infer past population size and distributional changes by evaluating the effect of different inference procedures on our results, comparing our palaeodistribution models to Pleistocene-aged packrat midden records, and simulating DNA sequence data under a variety of alternative demographic histories. Together the results indicate that these organisms have shared a common history of population expansion, and that these expansions were broadly coincident in time. However, contrary to our expectations, none of our analyses indicated significant range or population size reductions at the end of the last glacial period, and the inferred demographic changes substantially predate Holocene climate changes.

## Introduction

Comparative phylogeography seeks to understand how the geographic ranges of co-distributed species have changed over time [1]. By comparing population genetic patterns across species, it may be possible to discern whether changes in distribution and population size represent the influence of extrinsic factors that affected whole communities, or whether they can be ascribed to stochastic variation and other chance factors particular to each organism [2]. Several comparative studies have succeeded in identifying patterns common across many groups of organisms [3]–[5]. For example, phylogeographic studies of European terrestrial biota have found that many organisms, from insects [6], to mammals [7], to woody plants [8], show common patterns of Holocene range expansion from three common refugia [9]. Similarly, a large meta-analysis of organisms in southeastern North America found six common phylogeographic patterns repeated across many taxa [4].

More commonly, however, comparative studies reveal patterns that are idiosyncratic, with each species having experienced its own unique biogeographic history [10]–[15]. For example, in a comparative phylogeographic study of organisms occurring in the rocky-intertidal zone of the North Atlantic, Wares and Cunningham [16] found that although most species showed evidence of having recently colonized North American shores from Europe, the acorn barnacle, *Semibalanus balanoides*, persisted on the shores of both continents through the last glacial period. Similarly, in a series of papers Carstens and colleagues compared phylogeographic patterns across six species living in mesic forests of northwestern North America. All showed disjunct modern distributions spanning the Cascades and Rocky-Mountains, but genetic data suggest that these species achieved their current range in different ways. Some maintained separate populations in each mountain range throughout the last glacial, while others recently expanded from distinct glacial refugia [11], [17]–[19]. Even within communities showing largely concordant phylogeographic patterns, species displaying exceptional histories are frequent [4], [20]. In their meta-analysis of the spatial distribution of haplotypes across taxa in southeastern North America, Soltis and colleagues found that despite a few recurring themes overall phylogeographic patterns were not distinguishable from a random distribution of phylogeographic breaks, implying a complex history with little commonality across taxa [4].

The lack of concordance in community phylogeography is not restricted to studies of the spatial distribution of haplotypes, but extends to analyses of demographic history as well. A comparison of population histories in two sympatrically distributed terrestrial flatworms endemic to the Australian wet tropics found that although both of these organisms were extreme habitat specialists, Holocene climate changes were associated with quite different population size changes. One showed evidence of significant population expansions, while the other showed evidence of population declines [12]. Similarly, comparisons of ecologically similar, co-distributed skinks endemic to rainforests on the west coast of Australia found that climate change since the last glacial maximum had quite different demographic impacts across four species. *Saproscincus basiliscus* showed significant signatures of population expansions in northern populations, but no significant deviations from a constant population size in central and southern populations. Meanwhile the closely-related *S. tetradactyla*, *S. czechurai*, and *S. lewisi* showed either no evidence of population size change, or in the case of *S. tetradactyla*, expansion only in southern populations [10].

The disparate results across comparative phylogeographic studies present a quandary: why do some studies find congruent patterns across different members of the same ecological community, whereas others find strikingly dissimilar patterns from species to species? One possible explanation may be that geographic and topographic features of the landscape itself can in some instances limit the number of possible corridors for dispersal and the locations of potential refugia during glacial periods, which could lead to recurring phylogeographic patterns across taxa [4], [9]. Where there are many possible refugia and corridors for dispersal, congruent phylogeographic patterns may be less common. Alternatively, similarities and differences between intrinsic features of the organisms' biology, such as niche requirements and dispersal ability, may dictate the degree to which co-distributed species undergo similar range shifts over time [10], [17]. A third possible explanation, which has been proposed by several authors [15], [21], [22], is that the extent to which co-distributed species will share a common biogeographic history varies depending on the strength and specificity of ecological relationships between them. While generalist species that interact with one another only weakly may often have discordant biogeographic histories, organisms that are part of specialized or obligate interactions should frequently show shared distributions that persist through periodic environmental changes [15], [21], [23], [24].

Obligate pollination mutualisms, such as those between yuccas and yucca moths [25], or figs and fig wasps [26], [27], present an unusual opportunity to test the hypothesis that strongly interacting species should respond to extrinsic factors such as climate change in a concerted fashion. Within these systems, both the pollinators and the plants are mutually reliant upon one another for reproduction, and there is frequently a high degree of specificity. Among figs, although recent work has called into question the dogma that every species has its own unique pollinator, there is nevertheless a very high level of specialization, with roughly half of all figs reliant upon a single species of fig wasp for pollination [28]. In addition, where there are multiple pollinators per host the wasps are frequently close relatives or even sister species [29]–[32]. Similarly, among yuccas, of 27 species with known pollinators, 20 rely exclusively on a single species of yucca moth for pollination [33]–[35]. These systems also frequently host a diversity of non-pollinating associates that exploit the primary interaction, such as “bogus” yucca moths that oviposit into developing flowers and fruits without pollinating them [25], [36]–[39], and non-pollinating fig wasps that may feed on the developing fig, or may act as parasitoids that prey on the eggs and larvae of fig wasps [40]–[42].

**Figure 1:**
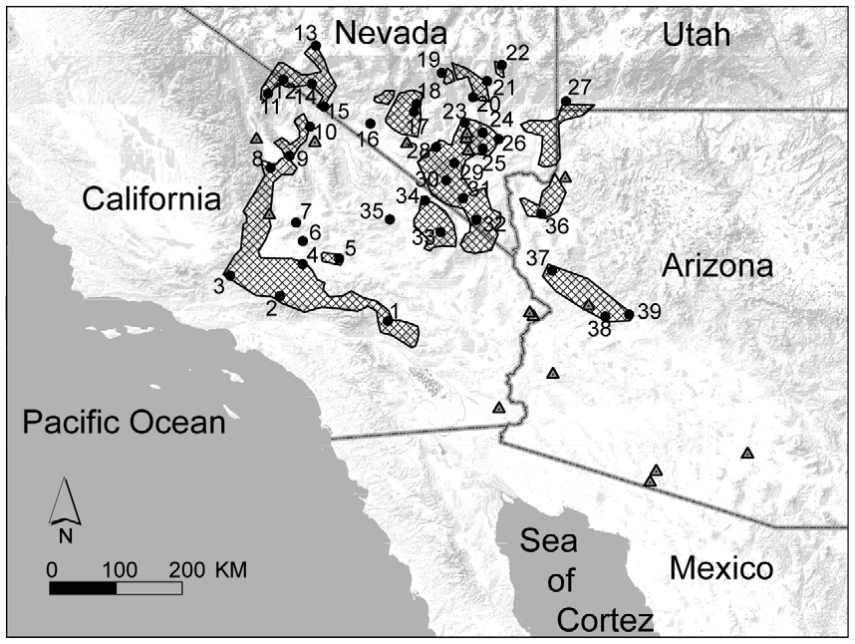
Study area showing the current distribution of *Y. brevifolia* (shaded areas), and the location of study sites (circles). The locations of 29 palaeorecords for *Y. brevifolia* from 13KYA or earlier are shown as grey triangles. Leaf tissue was sampled from all sites. Specimens of *Prodoxus sordidus* and *P. weethumpi* were sampled from sites 2, 13, 27, 33, and 37. Specimens of *Tegeticula antithetica*, which occurs in the eastern half of the range of *Y. brevifolia*, were collected from sites 19, 25, 27, 30, 33, and 37; specimens of *T. synthetica*, which occurs in the western of the range of *Y. brevifolia*, were collected from sites 2, 9, 11, 17, and 19.

One such system, the Joshua tree/yucca moth interaction, is ripe for an exploration of its shared biogeographic history. Joshua trees (*Yucca brevifolia*) are distributed across the Mojave Desert region of southwestern North America (Fig. 1), and are thought to have experienced significant range shifts in response to Quaternary climate changes [43]–[45]. Joshua tree leaves are frequently found in late Pleistocene midden records [46]–[48], and an extensive palaeorecord of past occurrences suggests that Joshua trees were distributed over a much larger geographic range than they are today. On this basis, some investigators have inferred that Joshua trees experienced large range and population contractions at the end of the last ice age [49], perhaps because the extinction of the North American megafauna reduced the plants' capacity to disperse and colonize new habitats. Joshua trees are pollinated by two distinct species of moths (*Tegeticula synthetica* and *T. antithetica*) [50], [51], and two sister species of non-pollinating “bogus” yucca moths parasitize the fruits and peduncles (*Prodoxus weethumpi* and *P. sordidus*, respectively) [52]. All four of these moths are specialists on Joshua tree, and together form a mutually interdependent community. There is, therefore, a strong *a priori* expectation that these species should have shared a common biogeographic history over time, and that signatures of this common history should be visible in genetic data obtained from each species.

However, the expectation of concerted changes is far from a foregone conclusion. First, as the Joshua tree is associated with two different pollinators across its range [50], an expansion of the range of the plant does not necessarily require that both insects expanded their distribution; newly founded populations of the plant could be associated with only one of the two pollinators. As a result, we might observe population expansions in one, but not both pollinators. Similarly, during range expansion a host plant may be able to temporarily escape specialist herbivores in newly-colonized habitats [53], [54]. So, we might expect asynchrony in range expansions, with expansion in parasitic species only occurring long after expansions in the host. Finally, stochasticity in the coalescent process might erase population genetic signatures of a shared biogeographic history.

Here, we present an integrated study of range size changes in the Joshua tree and four species of associated yucca moths. We used coalescent-based analyses of DNA sequence data to identify signatures of population size changes, including both summary statistics and parameter estimation. Next, we used species distribution modeling methods to infer the range of Joshua tree at the LGM (21 KYA), and compared these predictions to known palaeorecords to independently validate the reconstructed ranges. Last, to evaluate statistical support for our findings, we simulated DNA sequence data under alternative demographic histories and analyzed these data using these same coalescent-based methods, together producing more than 40 MB of simulated data and requiring more than 8000 hours of computer analyses. By comparing the empirical results with growth rates estimated from simulated data, we evaluated the power of our technique to distinguish alternative demographic histories.

## Methods

### Data collection

We collected plant leaf tissue and moths from multiple localities across the range of the Joshua tree, including multiple individuals per locality. Sampling included moths from five to six localities per species, and plant leaf tissue from thirty-nine localities (Figure 1 and TableS1). Differences in sampling intensity reflect the differences in genetic diversity within populations at sampled loci between Joshua tree and its insect associates. For each moth specimen, DNA sequence data were obtained from the mitochondrial genes cytochrome oxidase one (COI) (∼1400 bp) and nicotinamide adenine dinucleotide dehydrogenase subunit 5 (ND5) (∼400 bp), and the nuclear gene elongation factor one alpha (EF1α) (490 bp). Sequence data from all of these genes represent protein-coding regions exclusively. For each individual Joshua tree, DNA sequence data were obtained from five non-protein coding regions in the chloroplast genome, including the tRNA threonyl to tRNA leucine intergenic spacer (trnT-L), the tRNA leucine gene and intron (trnL and trnL intron), the tRNA leucine to tRNA phenylalanine intergenic spacer (trnL-F), and the caseinolytic peptidase, ATP-dependent, proteolytic subunit intron 2 (clpP). Genetic data from *Y. brevifolia*, *T. antithetica*, and *T. synthetica* were obtained from previous studies [51]; data from *P. sordidus* and *P. weethumpi* were obtained by PCR and thermal cycle sequencing using standard protocols.

Raw sequence data were visualized and edited using CodonCode Aligner v. 2.02 (CodonCode Corporation 2002–2007); putative mutations were identified both automatically using the “find mutations” feature in CodonCode Aligner, and manually by comparing aligned sequences. All putative mutations were confirmed by comparing electropherogram traces across fragments and across individuals. DNA sequences from protein-coding regions were translated to amino-acid sequences and checked to confirm that the sequences contained no stop codons. Unique mutations, or those resulting in non-synonymous substitutions were checked a second time against the original electropherograms. Preliminary alignments of sequences from within species were completed using the built-in algorithm in the CodonCode Aligner. Alignments to outgroup sequences (see below) were completed using MUSCLE [55]. MUSCLE alignments used default parameters, which have been shown to provide the best average accuracy in alignments [55].

Nuclear (EF1α) DNA sequences were scanned for heterozygosity using the automated mutation detection feature in CodonCode Aligner, and by manually double-checking electropherograms at sites with known mutations. Heterozygous genotypes were resolved using PHASE v. 2.1.2 [56], analyzing each species separately, using 10,000 iterations with a 1000 generation burn-in and a thinning interval of 10, and assuming no recombination. Chain convergence in the Markov Chain Monte Carlo analysis implemented in PHASE was assessed by comparing the posterior probability of all haplotype resolutions between two separate runs. Chain lengths were increased until no posterior probabilities differed by more than 0.01 between runs. Haplotypes that could not be resolved with >90% posterior probability in both of two independent runs were discarded.

The final genetic dataset included cpDNA sequence data for 79 individuals of *Y. brevifolia* (∼2 individuals per locality); mtDNA sequence data from 25 individuals of *P. sordidus*, 32 of *P. weethumpi*, 24 of *T. antithetica*, and 31 of *T. synthetic* (∼6 individuals per locality); and nuclear sequence data from 54 haplotypes from 27 individuals of *P. sordidus*, 58 haplotypes from 29 *P. weethumpi*, 43 haplotypes from 22 *T. antithetica*, and 49 allelotypes from 26 *T. synthetica* (∼6 individuals per locality) (See Tables S2 and S3). In *T. antithetica* and *T. synthetica* the number of phased haplotypes is less than twice the number of sampled individuals because the identity of both alleles from two genotypes could not be unambiguously resolved with >90% posterior probability.

### Analysis of molecular evolution

Models of sequence evolution for each gene region were selected under an Akaike Information Criterion using FindModel, a web implementation of Posada's ModelTest program [57], [58], hosted by the Los Alamos National Laboratory HIV Sequence Database, including all 26 possible models. Models were selected separately for each species, and each gene region, and the best-fit models were then used for estimation of population genetic parameters in BEAST and LAMARC. Selected models for each species and gene region are shown in Table 1. For the purposes of estimating rates of sequence evolution in EF1α (below), a single model (TIM+Gamma) was selected for sequences from all four moth species.

**Table 1:** Models of sequence evolution inferred using FindModel under AIC.

Species	Locus	Model Family	Kappa	Parameters in Rate Matrix	Rate Heterogeneity	Alpha
***P. sordidus***	mtDNA	TrN	Na	65.95, 24.28	Gamma-distributed rates	0.04
	EF1α	K81	2.01	Na	Gamma-distributed rates	0.04
***P. weethumpi***	mtDNA	HKY	25.90	Na	Gamma-distributed rates	0.04
	EF1α	K81	1.49	Na	None	Na
***T. antithetica***	mtDNA	TrN	Na	25.38, 14.72	Gamma-distributed rates	0.04
	EF1α	HKY	35.16	Na	None	Na
***T. synthetica***	mtDNA	HKY	43.26	Na	Gamma-distributed rates	0.04
	EF1α	HKY	39.72	Na	Gamma-distributed rates	0.04
***Y. brevifolia***	cpDNA	K81	2.81	Na	Gamma-distributed rates	1.55

Rates of sequence evolution were established based on previously published studies [51], [59], or were re-estimated for this study. Based on previously published rate estimates for these taxa [51], mutation rates per site in the mitochondrial genes were assumed to be 9×10^−9^ substitutions per site per year (S/S/Y) and 3.5×10^−8^ S/S/Y in the COI and ND5 genes, respectively, with an average of 1.5×10^−8^ S/S/Y across mitochondrial genes. Likewise, substitution rates within the *Y. brevifolia* chloroplast genome were assumed to be 7.6×10^−10^ S/S/Y and 1.1×10^−7^ insertions/deletions *per locus* per year following previously published rate estimates [51], [59]. Because previous rate estimates for EF1α in the Prodoxidae produced surprisingly high estimates (2×10^−8^ S/S/Y) we re-estimated mutation rates in this gene using a conventional molecular clock: phased sequence data from *P. sordidus*, *P. weethumpi*, *T. synthetica*, and *T. antithetica* were aligned to an outgroup sequence of *Lampronia rubiella* (Prodoxidae). The data set was sub-sampled, including one exemplar of each distinct allele. A model enforcing a molecular clock was compared to the best-fit model (TIM+Gamma), using a likelihood ratio test. Because the likelihood ratio test could not reject the molecular clock (*P* = 0.229, d.f. = 32), phylogenetic relationships among alleles were estimated by maximum likelihood enforcing a molecular clock, and using an heuristic search strategy with the starting tree estimated by neighbor joining and constraining the search to save no more than 400 trees. Rates of sequence evolution were estimated by setting the time to common ancestry of *Tegeticula* and *Prodoxus* to 29.91 MY, following previous studies [51], and estimating the number of substitutions that have accumulated in this time from the ML trees. This method produced a rate estimate of 2.2×10^−9^ S/S/Y. Rates of sequence evolution were identical in all 400 of the equally-likely topologies.

The potential role for natural selection in shaping substitution rates within protein coding genes (COI, ND5, EF1α) was assessed using a McDonald-Kreitman test [60] implemented in DNAsp v 5.10.01 [61], comparing the frequency of synonymous and nonsynonymous substitutions within species to those between species in *T. antithetica* and *T. synthetica*, and in *P. sordidus* and *P. weethumpi*, respectively. In no case was there evidence for statistically significant deviations from neutrality.

### Analysis of population structure

We tested for population structure within each species using an Analysis of Molecular Variation (AMOVA) [62], analyzing each gene region (plastid or nuclear DNA) from each species separately. AMOVAs used a standard haplotypic format; populations were grouped into regions according to one of two schemas (Northwest, Northeast, Central, Southeast, and Southwest, or North, Central, and South), and genetic variation was apportioned to differences between regions, differences between populations within regions, and differences within populations. Significance was assessed using 1000 permutations of the original data. Data sets were deemed to show evidence of significant population structure if global F_ST_ scores were greater than 0.2 and significantly different from zero.

### Analysis of demographic history

To test for changes in population size, we calculated summary statistics and estimated population genetic parameters associated with demographic change. Summary statistics were calculated using DNAsp version 5.10.01 [61]. Growth rates were estimated using LAMARC version 2.1.3 [63] and extended Bayesian skyline (EBSP) plots were constructed using BEAST version 1.5.3 [64], [65].

We calculated Fu's Fs [66], for each species and each locus, in DNAsp. Fs is expected to be negative, given a history of population expansion, or positive, given a history of population decline [67], and has greater power to detect population size changes than similar summary statistics, such as Tajima's D [66], [67]. Significant deviations from zero were assessed using coalescent simulations implemented in DNAsp [61], assuming a constant population size and a population mutation rate equal to that observed in the empirical data. To estimate the timing of population size changes, we used DNAsp to calculate τ, which is equal to the time since population size change measured in time units of 1/2 µ, where μ is the *per-locus* substitution rate [68].

Rates of population growth/decline relative to the neutral mutation rate (the parameter ‘g’) and genetic diversity (Θ) were estimated from sequence data using LAMARC v. 2.1.2b [63], using a maximum likelihood estimation procedure. Relative rates of sequence evolution between loci were based on absolute rate estimates inferred above. Models of sequence evolution were selected based on the FindModel results described above. Search strategies used ten initial short chains and 2 long chains per locus. Short chains were 2000 steps long, discarding the first 1000 steps, with a thinning interval of 20. Long chains were 40,000 steps long, discarding the first 1000 steps, with a thinning interval of 20. Chain convergence was assessed by comparing parameter estimates between two independent runs.

To estimate the magnitude and relative timing of population size changes across taxa, changes in the effective population size through time were reconstructed using extended Bayesian skyline plots (EBSPs) in BEAST version 1.5.3 [64], [65]. Strong population structure within a metapopulation can skew estimates of changes in the effective population size through time using skyline plots [69]; consequently for datasets in which significant population structure was identified, data were subsampled to include only one individual per deme. A strict molecular clock was enforced assuming substitution rates equal to the mutation rates described above (1.5×10^−8^ S/S/Y for the combined mtDNA, 2.2×10^−9^ for EF1α, and 7.6×10^−10^ S/S/Y for the combined cpDNA). Models of sequence evolution were selected based on the FindModel results described above. Tree priors used a coalescent tree assuming a stepwise model; starting trees were generated by UPGMA, and the ploidy of each gene region was set either to a mitochondrial or autosomal nuclear model as appropriate. The coefficient of variance and the covariance in rates of evolution were each assigned a normally-distributed prior, with means set to one and zero, respectively. Each dataset was analyzed using two separate Markov-Chain-Monte-Carlo (MCMC) simulations of 30 million generations in length. To ensure that the Markov Chains achieved convergence, effective sample sizes for each estimated parameter were computed using TRACER version 1.5, and the correlation in demographic parameter estimates between runs were compared in using commercially available spreadsheet software. If the correlation between runs was less 99%, or if Tracer identified that some parameters had unacceptably low effective sample sizes, run lengths were increased in 10 million generation increments.

To evaluate the statistical support for changes in population size, we counted the number of post-burn-in generations in which the inferred number of population size changes was greater than zero. The fraction of all post-burn-in generations in which the inferred number of changes was zero is equal to the posterior probability of no change in population size, given the data.

### Phylogeographic analysis

To evaluate evidence of geographic structure, we used a Mantel test [70] comparing geographic distance with F_ST_, implemented in Arlequin v. 3.5 [71]. For the Mantel test, great circle distances between populations were calculated using the Geographic Distance Matrix Generator v. 1.2.3. [72] and significance levels were calculated using 1000 permutations. If pairwise F_ST_ statistics were significantly correlated with geographic distances, and if global F_ST_ statistics were greater than 0.2 (indicating large divergence between populations [73]) datasets were then analyzed in a continuous phylogeographic analysis to infer changes in distribution over time. The phylogeographic analysis used a relaxed random walk with a one parameter gamma distribution model [74], and was implemented in BEAST v 1.6.1 [64]. Models of sequence evolution and priors on estimated parameters are as described for the extended Bayesian skyline analyses (above), and assumed a strict molecular clock based on previous estimates of substitution rates (see above). For the geographic distribution of species, a uniform prior was set using the coordinates for the most disjunct current or historical populations to set latitudinal and longitudinal boundaries on the prior. Analyses were completed as two independent MCMCs per data set, each of 800 million steps in length. Parameters were sampled every 50,000 steps, and the first 200 million generations were discarded as burn-in. Post-burn-in trees from each run were combined using LogCombiner v. 1.6.1 and summarized using TreeAnnotator v. 1.6.1. Changes in distribution through time, as inferred from the maximum clade credibility tree, were visualized using SPREAD 1.0 [75], and converted to GIS layers in ArcGIS v. 10.

### Distribution modeling

To provide a second line of evidence for changes in distribution and population size, we used distribution modeling to reconstruct the potential distribution of *Y. brevifolia* under current climate conditions, and at the LGM (21KYA), and then compared these distributions to infer changes in range and population size.

To validate the current range of Joshua trees and their associated moths, we compiled presence records from contemporary and historic sources. We consulted historic range maps [76], monographs [77], and species accounts [78], and obtained contemporary GIS records from the US Geological Survey's Digitized Range Maps for Modern Plants of the Southwest database [79]. These sources were then extensively ground-truthed; we visited every accessible population known from existing sources, recording GPS coordinates for true presence records (a tiny minority of populations located within restricted-access military reservations could not be visited; where possible GPS data for these sites were obtained from the responsible agencies). We supplemented these data with additional GPS records provided by the US Geological Survey, the Nevada Test Site, Joshua Tree National Park, The Mojave Desert Ecosystem Program, and Edwards Air Force Base. Together, these produced a dataset of 5765 GPS records for confirmed presences.

We compiled records of past occurrences of *Y. brevifolia* from the US Geological Survey Packrat Midden database. (Table S5). Radiocarbon dates for these records were converted to calendar years using the CALIB Radiocarbon Calibration Program 5.0.2 [80]. Of these records, we include only observations that are greater than 13,000 years old, the point when the distributions of many species in these regions began to change rapidly in response to climate change [43], [81].

Our climate models are based on the Worldclim dataset (Hijmans et al. 2005; http://www.worldclim.org/). These variables represent biologically meaningful summaries of precipitation and climate from the present (1950–2000). Richards et al. [82] provide hindcasting at 21,000 years BP for 14 of the first 19 Worldclim variables (See Table S4) using the CCM1 climatic projection [83] at a resolution of 2.5 arc minutes. Insufficient data were available to develop climate projections for the remaining five Worldclim variables.

We developed models of the potential distribution of *Yucca brevifolia* at the present and at the LGM using boosted regression trees (BRT) and maximum entropy (MaxEnt), two methods that performed very well in extensive comparison of available methods on empirical data [84]. We fit BRT in R [85] using Elith et al. 's [86] modifications of the GBM package [87], [88] with a tree complexity of 5, a learning rate of 0.001 and a bagging fraction of 0.5. Our MaxEnt analyses used MaxEnt V. 3.2.19 with default settings (logistic output, a regularization multiplier of 1, 500 iterations, a maximum convergence threshold of 0.00001 and a maximum of 10,000 background points). For each method we created a model by scoring the 212 2.5 arc minute grid cells for which there was at least one *Yucca brevifolia* occurrence as a presences and sampling pseudo absences (or background points) from locations within 500 km of the current and fossil range of *Y. brevifolia*. In BRT we used equal numbers of presences and pseudo absences (212). Our models used all 14 Worldclim provided in [82].

Distribution modeling methods are prone to make errors when extrapolating to non-analogous climates –environments that occurred in the past and have no equivalent in the current range of the study organism [89], [90]. To protect against this problem we computed the maximum and minimum value for each of our 14 variables in our current climate data set. We scored locations where the value of at least one variable was more extreme during the LGM than any value in our current climate dataset as non-analogous. We consider predicted presences in these regions as suspect (Figure S1).

We investigated the accuracy of predicted ranges (both today and at the LGM) using independent presence data. To determine the accuracy of our present day distribution models, we calculated Area Under Curve (AUC) statistics using a randomly selected portion of our data set that was not used to develop our models. This statistic ranges from zero to one, with a score of one indicating a model that perfectly distinguishes presences from absences and a model with a score of 0.5 indicating a model that performs no better than chance. We accomplished this in BRT by fitting ten separate models each including 90% of the available data and calculating AUC scores for each model with the remaining 10% [86]. We then present the AUC score averaged over all runs. In MaxEnt we re-ran our analyses using 90% of our data and present an AUC score for the remaining 10% of our current presences. In addition, we determined Cohen's kappa statistic [91], a measure of the agreement between two classification schemes (in our case predicted presences versus observed presences) that varies from zero to one. We calculated this statistic using datasets consisting of all presences and an equal number of randomly selected pseudoabsences. Using the same summary statistics (AUC scores and kappas) we measured the ability of each model to distinguish the thirteen grid cells from which we have pack rat midden fossils >13, 000 years old from an equal number of background cells.

Finally, to infer changes in distribution between the LGM and the present, we compared the number of cells in each time frame for which the probability of Joshua tree's presence exceeded a certain threshold. Since there is uncertainty about the best way to distinguish predicted presences from absences, we calculated two thresholds recommended by Lui et al. [92]. We first calculated two quantities, sensitivity –the proportion of correctly predicted presences– and specificity –the proportion of correctly predicted absences. We then determined the threshold that provides equal sensitivity and specificity and the threshold that maximizes the sum of specificity and sensitivity. MaxEnt calculates these quantities automatically. We used the PresenceAbsence package in R to calculate both thresholds for BRT [91]. We present predictions from each modeling method (BRT and MaxEnt) using data for the present climate, and for the climate during the LGM.

### Simulations

Some of the conventional methods for inferring past demographic changes from genetic data using either parameter estimation or the calculation of summary statistics have inherent directional biases [93]. Although the addition of data from multiple independent loci (as we have done here) is theorized to diminish the effect of this bias, it is unclear how much additional data is needed to overcome this bias. In order to evaluate the power of the techniques we have used here to infer past population size changes in the Joshua tree community, we expanded on the approach developed by Carstens et al. [18] by simulating coalescent trees and DNA sequence data under several alternative demographic histories, using models of sequence evolution selected based on the best-fit models inferred from the empirical data (Table 1). Contemporary effective population sizes were determined by dividing the empirical estimates of Θ estimated by LAMARC by twice the neutral substitution rate (Table 2). We used Mesquite v. 1.12 [94] to simulate data for each species, and each locus under each of the four possible demographic histories inferred from the palaeodistribution modeling (see Results): a constant population size through time (the history inferred using Boosted Regression Trees with equal sensitivity), a slight decline in population size (the history inferred using Boosted Regression Trees with maximum sensitivity), a slight increase in size (the history inferred using Maximum Entropy with maximum sensitivity), or a doubling in population size (the history inferred using Maximum Entropy with equal sensitivity).

**Table 2:** Haploid effective population sizes used for coalescent simulations.

Species	Locus	θ	mutation rate per site per generation	Ne
***P. sordidus***	mtDNA	0.03	1.50E-08	1.00E+06
	EF1α	0.09	2.20E-09	2.05E+07
***P. weethumpi***	mtDNA	0.04	1.50E-08	1.33E+06
	EF1α	0.01	2.20E-09	2.27E+06
***T. antithetica***	mtDNA	0.03	1.50E-08	1.00E+06
	EF1α	0.05	2.20E-09	1.14E+07
***T. synthetica***	mtDNA	0.03	1.50E-08	1.00E+06
	EF1α	0.04	2.20E-09	9.09E+06
***Y. brevifolia***	cpDNA	1.1	2.42E-08	2.27E+07

Values for Θ were estimated LAMARC v. 2.1.2b. Mutation rates are based on previously published values, and on new estimates described here, assuming one generation per year for the moths, and 30 years per generation for Joshua tree.

Most palaeoenvironmental studies for Mojave Desert region indicate that major range changes occurred between 13KYA and 9KYA [43], [44], [95]. Therefore, simulated data sets assume a constant population size until 13KYA, followed by a single, instantaneous population size change, and then a constant population size from 13KYA to the present. We assumed one generation per year for the moths, and 30 years per generation for *Y. brevifolia*.

Simulated data were then analyzed using LAMARC and BEAST, as above, and VariScan [96], a command-line-based, scriptable software that completes many of the same analyses contained in DNAsp. Signatures of population growth in the empirical data were then compared with those expected under these alternative demographic histories. The frequency of simulated data sets showing signatures of population growth as great, or greater than those seen in the empirical data is the probability of obtaining the observed value, given a particular demographic history.

## Results

### Analysis of population structure

For all insect species and gene regions population structure was weak overall, with local populations containing between 80% and 97% of the total genetic variation, and with average F_ST_ scores of 0.12, indicating moderate divergence through genetic drift [73] (Table 3). However, the F_ST_ and AMOVA scores were statistically significant in most cases, indicating that the apparent population structure cannot be attributed to sampling variance. The results of the population structure analyses in the plants differed markedly, however, from those seen in the insect data sets; less than 13% of the total genetic variation was contained within populations, and more than 56% was distributed among regions. Global F_ST_ was 0.87, suggesting very great divergence [73] (Table 3).

**Table 3:** Results AMOVAs calculated in Arlequin v. 3.5, by species and by locus.

*P. sordidus* mtDNA							
Source	df	Sum of Squares	Variance Component	% Variation	Index	Score	P
Among Regions	2	4.311	−0.075	−4.82	FCT	−0.04821	0.675
Among Populations Within Regions	2	5.056	0.304	19.48	FSC	0.18588	0.063
Within Populations	16	21.300	1.33	85.34	FST	0.14663	**0.006**
Total	20	30.667	1.56				

*P. sordidus* EF1α							
Source	df	Sum of Squares	Variance Component	% Variation	Index	Score	P
Among Regions	2	0.905	0.008	2.41	FCT	0.0241	0.331
Among Populations Within Regions	2	0.63	0.000	−0.13	FSC	−0.0013	0.340
Within Populations	49	15.650	0.319	97.72	FST	0.02283	0.290
Total	53	17.185	0.327				

*P. weethumpi* mtDNA							
Source	df	Sum of Squares	Variance Component	% Variation	Index	Score	P
Among Regions	2	5.134	−0.264	−11.55	FCT	−0.11546	0.930
Among Populations Within Regions	2	8.504	0.49576	21.72	FSC	0.19473	0.391
Within Populations	20	41.002	2.051	89.83	FST	0.10175	**0.030**
Total	24	54.64	2.282				

*P. weethumpi* EF1α							
Source	df	Sum of Squares	Variance Component	% Variation	Index	Score	P
Among Regions	2	0.797	−0.037	−10.67	FCT	−0.10674	0.722
Among Populations Within Regions	2	1.924	0.062	17.95	FSC	0.16216	**0.034**
Within Populations	53	17.055	0.321	92.73	FST	0.07273	0.059
Total	57						

*T. antithetica* mtDNA							
Source	df	Sum of Squares	Variance Component	% Variation	Index	Score	P
Among Regions	2	12.028	−0.029	−0.9	FCT	−0.00896	0.462
Among Populations Within Regions	2	10.165	0.666	20.82	FSC	0.2063	**0.005**
Within Populations	19	48.724	2.564	80.08	FST	0.19919	**0.005**
Total	23	70.917	3.202				

*T. antithetica* EF1α							
Source	df	Sum of Squares	Variance Component	% Variation	Index	Score	P
Among Regions	2	1.518	0.027	10.08	FCT	0.10076	0.138
Among Populations Within Regions	2	0.674	0.0182	6.686	FSC	0.07626	0.381
Within Populations	36	7.955	0.221	83.07	FST	0.16934	**0.006**
Total	40	10.146	0.266				

*T. synthetica* mtDNA							
Source	df	Sum of Squares	Variance Component	% Variation	Index	Score	P
Among Regions	2	10.239	−0.679	−19.44	FCT	−0.1944	0.700
Among Populations Within Regions	2	19.604	1.306	37.42	FSC	0.31331	**0.001**
Within Populations	24	68.708	2.863	82.02	FST	0.17982	**0.000**
Total	28	98.552					

*T. synthetica* EF1α							
Source	df	Sum of Squares	Variance Component	% Variation	Index	Score	P
Among Regions	2	3.443	0.055	5.84	FCT	0.05838	0.195
Among Populations Within Regions	2	1.896	0.006	0.67	FSC	0.00714	0.273
Within Populations	44	39.069	0.88793	93.49	FST	0.0651	0.064
Total	48	44.408	0.94976				

*Y. brevifolia* cpDNA							
Source	df	Sum of Squares	Variance Component	% Variation	Index	Score	P
Among Regions	4	36.468	0.56	56.28	FCT	0.56284	**0.000**
Among Populations Within Regions	32	24.216	0.307	30.83	FSC	0.70515	**0.000**
Within Populations	39	5.000	0.128	12.89	FST	0.8711	**0.000**
Total	75	65.684	0.995				

Populations were grouped into regions into three or five regions (North, Central, and South; or Northwest, Northeast, Central, Southwest, and Southeast).

### Analysis of demographic history

**Figure 2:**
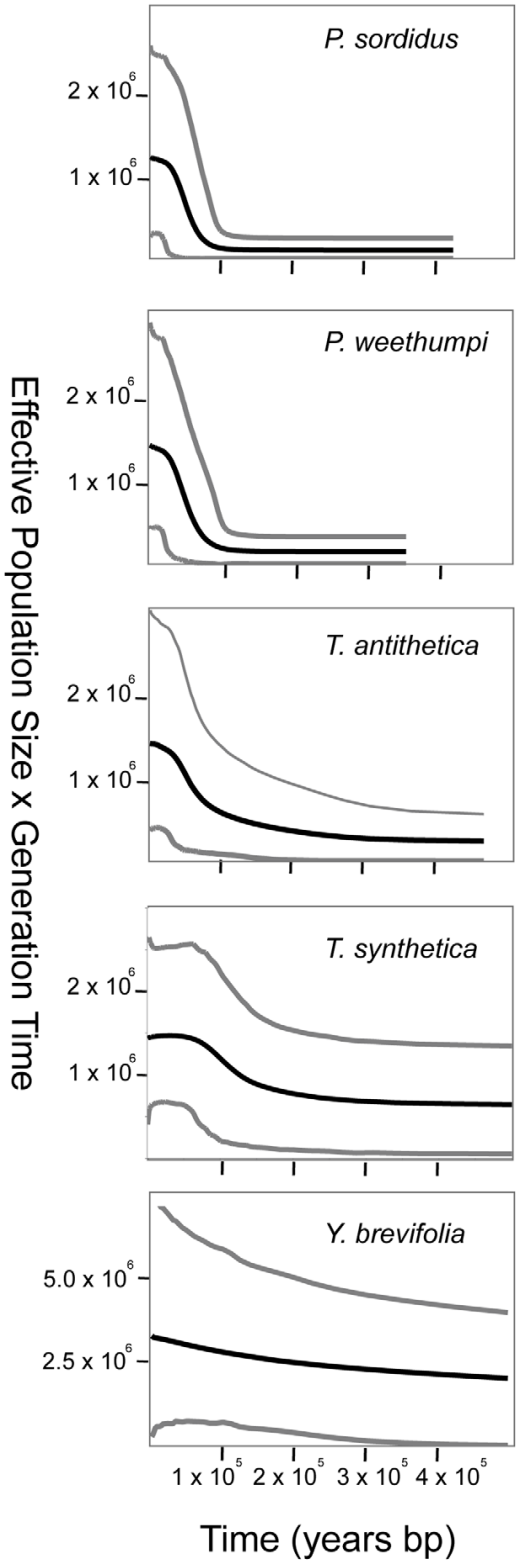
Extended Bayesian Skyline Plots inferred using BEAST v. 1.5.3 from DNA sequence data for *Yucca brevifolia* and four species of associated yucca moths. The dark lines show the mean of the highest posterior density (HPD) function of population size at each point in time, the grey lines show the upper and lower 95% credibility intervals on the HPD, averaged across multiple independent Markov Chain Monte Carlo simulations. Parameter estimates were >99% correlated between independent runs. Note that the y-axes are not to scale.

Estimates of population growth parameters (Tables 4, 5) indicate large population growth in *Y. brevifolia* and all four of the moth species. In all species the growth rates ‘g’ estimated by LAMARC were significantly positive (Table 4). Fu's Fs had large negative values for all species and all loci (Table 5), and these values were significantly different from those expected under a constant-size model for all datasets except EF1α from *P. weethumpi*, which was not significant (*p = 0.107)*. The inferred number of population size changes in post-burn-in samples from the EBSP analyses suggests that the hypothesis of demographic constancy (i.e. no change in population size) for can be rejected for *P. sordidus*, *P. weethumpi*, and *T. antithetica* (*p = 0.008*; *0.004*; *and 0.038*, respectively), but this hypothesis cannot be rejected for *T. synthetica* and *Y. brevifolia*, (*p = 0.222* and *0.375*). The EBSPs suggest that this population growth was largely coincident in time across species, beginning between 100 and 200 KYA, and continuing through 10 to 30 KYA (Fig. 2). These estimates indicate that population growth in this community may have begun long before the end of the last glacial period. The age of population size changes as estimated by Rogers and Harpending's *τ* varied considerably by locus and by species; for the moths age estimates varied between 15 and 200 KYA, but for the Joshua tree the estimated age of population size changes was 458 KYA (Table 6).

**Table 4:** Empirical estimates of population growth rates (scaled relative to the neutral mutation rate) and the population genetic parameter Θ estimated in LAMARC v. 2.1.2b [63].

LAMARC Results					
***P. sordidus***			***P. weethumpi***		
Gene	ML Growth Estimate(95% CI)	Θ (95% CI)	Gene	ML Estimate (95% CI)	Θ (95% CI)
COI	2839.37 (964.07, 7207.96)	0.03 (0.01, 0.23)	COI	2251.80 (887.55, 4530.71)	0.04 (0.01, 0.15)
EF1α	1836.08 (655.77, 2073.37)	0.09 (0.02, 0.18)	EF1α	392.78 (−82.77, 1807.34)	0.01 (0.01, 0.02)
Both	1411.94 (657.45, 1592.00)	0.01 (0.005, 0.02)	Both	1505.50 (983.65, 1991.66)	0.01 (0.006, 0.01)
***T. antithetica***			***T. synthetica***		
Gene	ML Growth Estimate(95% CI)	Θ (95% CI)	Gene	ML GrowthEstimate (95% CI)	Θ (95% CI)
COI	901.21 (100.67, 1971.11)	0.03 (0.01, 0.23)	COI	912.08 (288.80, 1905.96)	0.03 (0.01, 0.07)
EF1α	1534.89 (137.88, 2557.40)	0.05 (0.005, 0.18)	EF1α	221.50 (103.028, 461.58)	0.04 (0.03, 0.30)
Both	1674.15 (858.60, 2262.47)	0.02 (0.01, 0.03)	Both	248.56 (157.21, 328.30)	0.02 (0.01, 0.04)
***Y. brevifolia***					
Gene	ML Growth Estimate(95% CI)	Θ (95% CI)			
cpDNA	20.04 (5.23, 24.61)	1.12 (0.55,1.56)			

**Table 5:** Fu's Fs values calculated from empirical and simulated data sets by species and by locus.

Empirical				Proportion of Simulated Data Sets with Fs<Observed			
Species	Locus	Fs	P	Decline	Constant	Slight Growth	Doubling
*P. sordidus*	mtDNA	−8.206	*p = 0.001* *	0.041**	0.074	0.036**	0.057
	EF1α	−3.492	*p = 0.017* *	0.430	0.610	0.390__	0.370
*P. weethumpi*	mtDNA	−10.977	*p = 0.000* *	0.002**	0.003**	0.008**	0.011**
	EF1α	−2.012	*p = 0.107*	0.240	0.190	0.389__	0.112
*T. antithetica*	mtDNA	−7.598	*p = 0.003* *	0.031**	0.030**	0.038**	0.047**
	EF1α	−3.306	*p = 0.012* *	0.120	0.100	0.090__	0.090
*T. synthetic*	mtDNA	−7.882	*p = 0.002* *	0.024**	0.026**	0.030**	0.053**
	EF1α	−9.312	*p = 0.000* *	0.380	0.300	0.220__	0.460
*Y. brevifolia*	cpDNA	−3.848	*p = 0.035* *	0.114	0.110	0.134__	0.134

*Significant based on simulations implemented in DNAsp.

**Significantly different from empirical data.

Values for empirical data (left) were calculated in DNAsp v. 5; significance values are based on simulation in DNAsp assuming a constant population size and sequence variation equal to that seen in the empirical data. Values of Fs were also calculated for datasets simulated in Mesquite v. 1.12 (right) under a demographic scenarios inferred from distribution modeling and using models of sequence evolution (Table 1) and effective population sizes (Table 2) inferred from the empirical data.

**Table 6:** Tau calculated from empirical data in DNAsp v. 5, by species and by locus.

Species	Locus	Tau	Mutations/locus/year	time since population size change (years)
*P. sordidus*	mtDNA	0.462	3.01×10^−5^	1.53×10^4^
	EF1α	0.648	8.82×10^−6^	7.35×10^4^
*P. weethumpi*	mtDNA	2.954	3.01×10^−5^	9.81×10^4^
	EF1α	0.32	8.82×10^−6^	3.63×10^4^
*T. antithetica*	mtDNA	3.936	3.01×10^−5^	1.31×10^5^
	EF1α	0.52	8.82×10^−6^	5.90×10^4^
*T. synthetica*	mtDNA	3.072	3.01×10^−5^	1.02×10^5^
	EF1α	1.85	8.82×10^−6^	2.10×10^5^
*Y. brevifolia*	cpDNA	0.862	*1.88×10^−6^	4.58×10^5^

*Includes rates of substitution and insertion/deletion.

Mutation rates are based on published per-site rates, and rates estimated here, multiplied by the number of sites in each dataset.

### Phylogeographic Analysis

For most species/gene combinations the Mantel tests indicated no association between population differentiation and geographic distance, but in the Ef1a dataset from *T. antithetica* there was indication of a significant correlation (R^2^ = 0.581; *p = 0.009*) between geographic distance and pairwise F_ST_ scores (Table 5). As with the AMOVA results above, the results of the Mantel tests for the chloroplast were quite different from those for the insects. In the cpDNA data the global F_ST_ score was remarkably high (0.871), and the Mantel test revealed a highly significant correlation between F_ST_ score and geographic distance (R^2^ = 0.378; *p<0.001*) (Table 7).

**Table 7:** Results of Mantel tests comparing geographic distance with pairwise F_ST_ scores between populations, estimated in Arlequin v. 3.5.

*P. sordidus* mtDNA		*P. weethumpi* mtDNA	
R^2^	0.134	R^2^	0.0989
P	0.484	P	0.498

*P. sordidus* EF1α		*P. weethumpi* EF1α	
R^2^	−0.050	R^2^	0.186
P	0.580	P	0.371

*T. antithetica* mtDNA		*T. synthetica mtDNA*	
R^2^	−0.120	R^2^	−0.171308
P	0.540	P	0.625

*T. antithetica* EF1α		*T. synthetica* EF1α	
R^2^	0.581	R^2^	0.520
P	**0.009**	P	0.075

*Y. brevifolia cpDNA*			
R^2^	0.378		
P	**0.000**		

**Figure 3:**
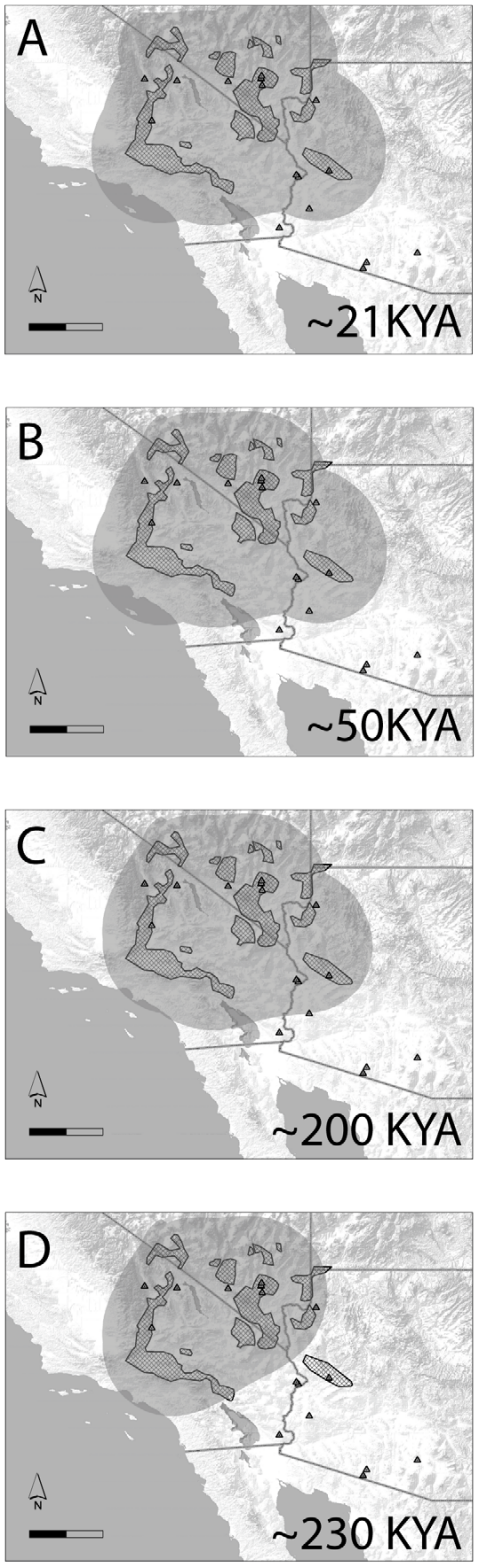
Spatial distribution of cpDNA haplotypes from *Y. brevifolia* through time. Distribution changes were inferred in a continuous phylogeographic analysis using a relaxed random walk with a one parameter gamma distribution model implemented in BEAST v. 1.6.1. Grey shading represents the 80% highest posterior density regions; that is, uncertainty about the location of internal nodes in a phylogeny of cpDNA haplotypes. The timing of distribution changes are based on rates of sequence evolution in cpDNA, assuming a strict molecular clock. Scale bars are 200 km.

Only the *Y. brevifolia* cpDNA data showed both significant population structure and a significant correlation of genetic distance with geography. Phylogeographic analysis of these data in BEAST revealed an origin in the central Mojave Desert region (south-central California), followed by range expansions into the edges of the Sonoran (western Arizona) and Great Basin (central Nevada) Deserts approximately 200 KYA, coincident with the demographic expansions seen in the Extended Bayesian Skyline Plots (Figure 3). An animation of the inferred distribution changes through time, is available as a .KML file viewable in Google Earth (File S1).

### Species distribution models

**Figure 4:**
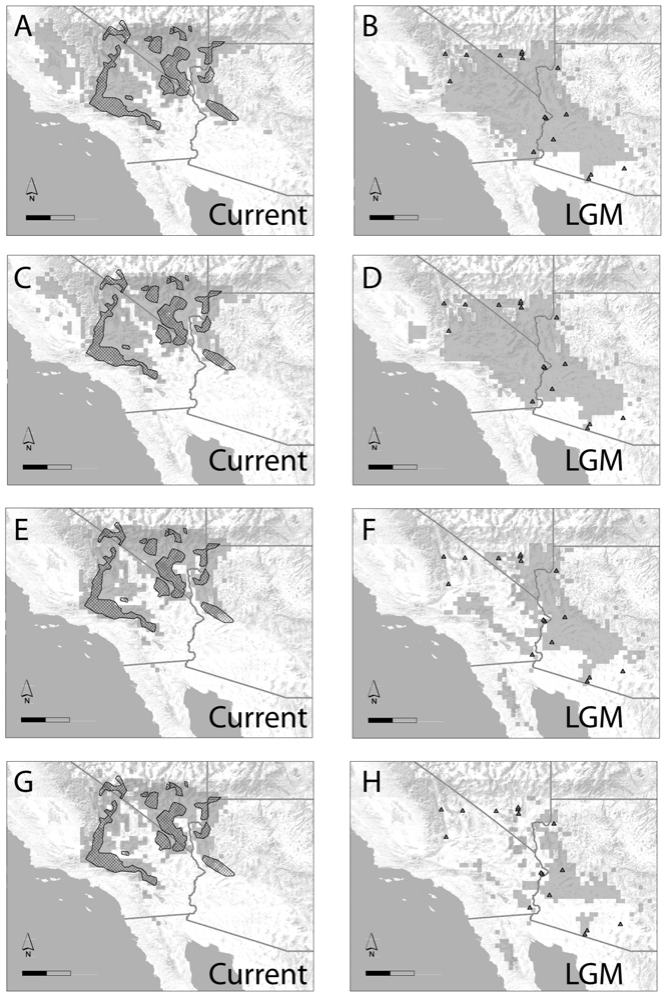
Estimated potential distribution for *Yucca brevifolia* (shaded cells) under current climate conditions (left column), and at the last glacial maximum (LGM) (right column). Distributions were inferred using boosted regression trees (panels A–D) and maximum entropy (panels E–H). For each algorithm, thresholds were set at values that either maximized sensitivity and specificity (panels A–B and E–F), or where sensitivity and specificity were equal (panels C–D and G–H). For comparison, the locations of 29 palaeorecords for *Y. brevifolia* from 13KYA or earlier are shown as grey triangles; the actual current range of *Y. brevifolia* is shown as cross-hatched polygons. Scale bars are 200 km.

All models inferred moderate changes in potential distribution, but the specific change varied with the algorithm and threshold used. Boosted Regression Tree (BRT) models predicted some probability of presence for *Y. brevifolia* across the entire Mojave Desert region (Figure 4). Using a threshold with equal sensitivity and specificity (predicted presence if probability >0.605) the distribution of *Y. brevifolia* contracted slightly (566 current vs. 641 past presences). Using a threshold that maximizes sensitivity and specificity (predicted presence if probability >0.55), the range changed very little (657 current vs. 693 past). The Maximum Entropy (MaxEnt) models predict that the trees were present in the southeastern portion of the Mojave Desert with a high probability, but indicate a low probability of presence in the remainder of the Mojave. Using a criterion of equal sensitivity and specificity (cutoff of 0.308) approximately twice as many locations are now suitable as were in the past (432 vs. 240). Using a threshold that maximizes the sum of specificity and sensitivity (0.224), slightly more locations are currently suitable than at the last glacial maximum (509 vs. 434).

Despite the differences between the various models, there are several points of agreement. All of the reconstructed palaeodistributions suggest that Joshua trees formerly occupied a much larger range in the southern Mojave, and show support for past occurrences in southern Arizona (where *Y. brevifolia* is known from palaeorecords, but is absent in modern assemblages). All of the reconstructed distributions also suggest that extinctions in the south seem to have been offset by localized range expansions in the north, as none of the reconstructed palaeodistributions showed evidence for dramatic declines in the total number of presences (contra [45]).

BRT and MaxEnt both accurately predicted the current distribution of *Y. brevifolia* (Figure 4). BRT models had an Area Under Curve (AUC) score of 0.92, while MaxEnt had a score of 0.952. For MaxEnt, Cohen's kappa statistic was 0.61 for the equal sensitivity and specificity criterion, and 0.71 for the maximum specificity plus sensitivity criterion, representing substantial agreement between predicted and observed presences [97]. For BRT the kappa statistic was 0.85 for both thresholds, representing a near perfect agreement [97].

All measures of accuracy were markedly lower for hindcasting than they were for predicting current presences. Using either threshold method, BRT models predicted presences in 7 of the thirteen grid cells containing known palaeorecords. These models had an acceptable AUC score of 0.805+/−0.087, and Kappa scores of 0.45 and 0.38 for equal sensitivity and specificity and maximized sensitivity plus specificity, respectively. Our MaxEnt model predicted four presences correctly using the equal sensitivity and specificity criterion (Kappa = 0.31; fair agreement), and six presences correctly using the maximum sensitivity plus specificity criterion (Kappa = 0.46; moderate agreement). This model had and had an AUC score of 0.76+/−0.1.

### Simulations

**Figure 5:**
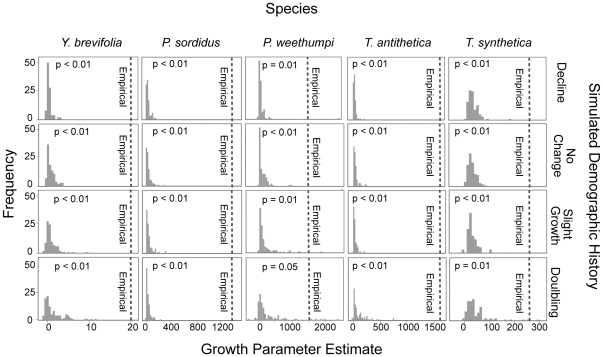
Estimated growth rates in simulated DNA sequence data sets under each of four alternative demographic histories. DNA sequence data were simulated in Mesquite v. 1.12 using models of sequence evolution, mutation rates, and current effective population sizes inferred from the empirical data for each species. Growth rate parameters were estimated in LAMARC v. 2.1.2b. Dashed lines show the empirically estimated growth rate parameter for each species; p values show the probability of observing values as large, or larger under each demographic scenario.

Results of the analyses of simulated data are shown in Figure 5, Table 5, and as Figure S2. For all five species, the growth rates estimated by LAMARC from the empirical data were significantly greater (*p*<0.01) than those inferred from data simulated under histories of population decline, constant population size, or slight growth (Figure 5; Table S6). However, under a history of population doubling a minority of the simulated data sets showed signatures of population growth comparable to those seen in the empirical data. Fu's Fs scores calculated from empirical data were, in most cases, significantly different from the simulated mtDNA datasets, but not the simulated EF1α data (Table 5), suggesting that the signatures of population expansion are greater in the mitochondrial data than in nuclear data. The EBSPs inferred from the empirical data indicated population growth that was of considerably greater magnitude than that seen in any of the simulated data (Figure S2). Together, these results strongly suggest that the common genetic signatures of population growth seen in Joshua trees and their associated yucca moths are unlikely to have been generated by chance, or by biases in the inference procedure.

However, the simulations also suggest that our data have relatively weak power to distinguish between the alternative demographic histories inferred from distribution modeling. In the simulated data, the distributions of the growth parameter estimates were all clustered near zero (Figure 5), and the average growth rates in the simulated data sets differed little between alternative demographic histories (although the distributions of estimated growth parameters were more right-skewed under the large growth scenario). Similarly, in comparing the EBSPs inferred from the simulated data (Fig. S2), the shapes of these plots are only very slightly different between alternative demographic histories. Thus, the true histories of these organisms likely involved demographic events of much greater magnitude than were simulated here; moderate changes in population size do not leave large enough signatures in the genetic data to be identifiable.

In addition, the Bayesian skyline plots seemed to have poor ability to precisely infer the timing of demographic changes (Fig. S2). Whereas the simulated data were generated under histories of population size change at exactly 13KYA, the Bayesian skyline plots indicate size changes beginning anywhere from 50KYA to 200KYA. Thus, it is difficult to determine whether the apparent onset of population growth prior to the end of the last glacial period inferred from the empirical data truly reflects demographic events in the distant past, or whether it may be an artifact of the relatively low power in our data.

## Discussion

Joshua tree and all four of its associated yucca moths show genetic signatures of large, concerted population growth during the late Pleistocene, and population growth was broadly contemporaneous with expansion of the Joshua tree's range. The signatures of concerted population growth in the trees and in the moths, together with phylogeographic signatures of range expansion likely reflect expansion of this entire community into the Sonoran and Great Basin deserts, from the Mojave Desert, beginning ∼200KYA. The common population size changes in these species suggest that groups of mutually specialized organisms may be likely to experience common range and population size changes over time, responding in congruent fashions to extrinsic geological and climatological changes.

However, the genetic signatures of growth are significantly greater than the range size changes inferred from palaeodistribution modeling, and the inferred growth appears to have occurred prior to the end of the last glacial period. This may indicate that Holocene climate change had little impact on the total population size of the Joshua tree and its associated insects. Although palaeorecords clearly indicate that Joshua trees occurred over a much broader geographic area during the last glacial period [45], we found no indication of dramatic population declines in *Y. brevifolia* since the LGM. The distribution models also suggest that the total potential distribution was either constant, or increased slightly between the LGM and today, and that habitat loss in the southern part of the Joshua tree's range was offset by the addition of new potential habitats in the north.

Similarly, we found no evidence that the extinction of the North American mega-fauna caused changes in the rates of dispersal in Joshua trees, as has recently been suggested [45]. First, had the extinction of large mammals caused reductions in total dispersal rates, we would expect to see signatures of dramatic population declines associated with the extinction of southern populations and reduced capacity to disperse to nearly available habitats in the north. The genetic data do not support recent population size reductions (indeed the evidence suggests significant population growth, rather than decline, albeit long before the Holocene). Second, had Joshua trees experienced significant recent declines in dispersal ability, we should expect to see large areas of potential distribution where the current climate is suitable, but where *Y. brevifolia* is absent due to dispersal limitation. Instead, comparisons between the predicted distribution and actual range of the Joshua tree indicated substantial (MaxEnt) to nearly perfect (BRT) agreement between the predicted and observed range.

### Comparisons of historical demography across loci and across species

Whereas all of the data analyzed here suggest similar patterns of population growth within the last two hundred thousand years, there is appreciable variation in both the magnitude of, and statistical support for population growth across species, loci, and analytical methods. In general statistical support for population growth was greatest in the mtDNA data, but appreciably weaker in the EF1α data, and very weak in the cpDNA data. Although the LAMARC analyses inferred growth rates significantly greater than zero for all species in the analyses of the combined data, signatures of growth were not statistically significant in the *P. weethumpi* EF1α data. Similarly, whereas evidence for population expansion based on summary statistics (Fu's Fs) were all statistically significant for mtDNA data, the signatures of growth were not significant for the *P. weethumpi* EF1α data, and for none of species were the Fs values for EF1α significantly lower those calculated from simulated data. Finally, although signatures of population growth contained in the cpDNA data were statistically significant, none were significantly lower than those calculated from simulated data, and the EBSP analysis did not show statistically significant support for changes in population size.

The lower statistical support for population growth in the Fs values calculated for the chloroplast and moth nuclear data may be attributable to the low power of summary-statistics (relative to parameter estimation) to infer population growth. The relatively small amount of variation contained in the EF1α and cpDNA datasets, may also have played a role. Both of these genetic markers have lower rates of sequence evolution than the mtDNA; as the amount of sequence variation in the data declines, the power to distinguish alternative demographic histories decreases accordingly. Finally, differences in the effective population size of the mitochondrial and nuclear genomes may have contributed to the stronger signatures of population growth seen in the mtDNA. Simulation work has shown that population bottlenecks result in greater reductions in genetic variation and larger signatures of population growth in the mitochondrial genome than in nuclear data [98].

### Phylogeographic patterns

As with the demographic analyses, above, there was considerable variation in the degree of and statistical support for population and geographic structure in the data. For the moths F_ST_ values and significance of AMOVAs were always lower in the EF1α data than in the mtDNA, perhaps reflecting male-biased dispersal, which has been identified previously within the Prodoxidae [99], or perhaps due to the lower sequence variation in the EF1α data. Similarly, whereas there was little geographic structure within the moth data, there was significant structure in the *Y. brevifolia* cpDNA data; F_ST_ scores indicated very great divergence between populations and were significantly correlated with geographic distance. That we find strong geographic structure in the cpDNA data but not in the moths is perhaps unsurprising given the inherently high dispersal ability of winged insects and the known low rates of seed dispersal in *Y. brevifolia* [100]. Geographic signatures of range expansion might therefore have been erased by subsequent dispersal in the moths, but not in the plants.

Phylogeographic analysis of the cpDNA data indicated range expansion into Sonoran Desert around 200KYA, contemporaneous with the onset of population growth seen in the pollinators, and that the Joshua trees achieved an essentially modern distribution by 50KYA (Figure 3). However, the phylogeographic analysis does not show evidence of past occurrences of Joshua trees in extreme southern Arizona, despite fossil records documenting the occurrence in these areas. This discrepancy is almost certainly due to the lack of DNA samples from these (now extinct) populations; in the absence of data recording the past occurrences in these areas, the analysis has no means to infer them. However, it seems likely that Joshua trees colonized these areas around 200KYA, coincident with their arrival in other Sonoran Desert populations.

### Timing of demographic changes

Estimates of the timing of the inferred population expansions suggest that they were largely contemporaneous (i.e., between 100 and 200 KYA) across all organisms, but the low precision of the age estimates make it difficult to definitively identify exactly when the onset of population growth occurred in each species. Inspection of the EBSPs (Figure 2) suggests that growth began approximately 200KYA in the trees and the pollinating moths (*T. antithetica* and *T. synthetica)*, and approximately 100KYA in the bogus yucca moths (*P. sordidus* and *P. weethumpi*).

The estimates of the timing of demographic changes based on Rogers and Harpending's τ statistic were fairly consistent with these estimates for the moths, but not for plants (Table 6). Values of τ for the pollinators suggest an onset of growth between 50KYA and 200KYA, and for the bogus yucca moths they suggest growth beginning between 15KYA and 98KYA. However, for *Y. brevifolia* the τ statistic suggests growth beginning ∼460KYA, well before the onset of growth seen in the EBSP and continuous phylogeographic analysis. (This value does correspond to the time to common ancestry of each of the two major haplotype lineages recovered in the EBSP of the cpDNA data (gene tree not shown)). It is important to note, however, that the range of ages inferred from the τ statistic reflect differences in rates of molecular evolution across loci, not statistical confidence intervals. The actual precision with which this approach can resolve the timing of demographic changes is not known.

### Comparisons of population genetic and palaeodistribution data

Although the population genetic data offer a fairly consistent view of population size changes in the Joshua tree – yucca moth community, there is notable discord between the results of the genetic data analysis and the palaeodistribution estimates. Signatures of population growth in the genetic data are appreciably greater than the changes in range size inferred from the palaeodistribution modeling (cf. Figures 2 and 4). Whereas the reconstructed ranges suggest that the distribution of Joshua tree grew only slightly, the EBSPs suggest that the trees' population size has at least doubled, and that the population sizes of the associated moths have grown between three- and ten-fold. The growth rate parameters estimated from the empirical genetic data were also larger –by an order of magnitude in most cases– than those seen in simulated data. Indeed, the probability of observing signatures of population growth as great as those seen in the data given the histories inferred from the distribution modeling is generally less than 0.01 (Figure 5).

That the signatures of population growth in the genetic data are so large could indicate that newly available habitats were colonized by individuals from a subset of the previously existing populations, perhaps on the northern periphery of the range, as has been seen in other cases of post-glacial expansion [9], [101]. Data simulations have shown that this ‘leptokurtic’ model of range expansion can give rise to very large signatures of population growth [6]. This explanation seems unlikely, however, as *Yucca brevifolia* does not seem to have undergone a large northward expansion overall – Joshua trees were present in a number of areas near the current northern limit of the species range even at the last glacial maximum. Last, it may be that our distribution models do not accurately reflect the total population size at either the LGM, at the present, or both. Distribution modeling reconstructs only the potential habitats that are likely to have been suitable given the climate; if dispersal limitation excluded trees from some potential habitat, either in the past or in the present, this difference would not be captured in the distribution modeling. Similarly, changes in population density, without concomitant changes in range size, might explain the discrepancy between the population genetic data and the reconstructed palaeodistributions.

Distinguishing signatures of population growth from selective sweeps is a well-known and vexing problem for inferring demographic histories from sequence data [66], [102]. Indeed, it has been argued that the evolution of the mitochondrial genome may be dominated by selective sweeps and genetic hitchhiking [103]. Natural selection might therefore account for the discord between the population genetic and palaeodistribution data. We reject this possibility for three reasons. First, comparisons among independently assorting loci can help to distinguish demographic expansion from positive selection [104]; that we find signatures of population expansion in both the mitochondrial and nuclear data (though only two loci were sequenced) argues for demographic expansion over positive selection. Second, comparisons of the number of synonymous and non-synonymous substitutions within and between species using a MacDonald Kreitman test did not did not reveal significant deviations from neutrality. Though selection acting elsewhere in the mitochondrial genome, which is non-recombining, would not be detected by this method, this test should have found evidence for selective sweeps in the nuclear data, had they occurred. Finally, it seems exceptionally unlikely that selection acting independently in each species would have produced the signatures of *contemporaneous* population growth seen here.

The simplest explanation for the discord between the genetic and paleodistribution data may therefore be merely that large population expansions occurred prior to the end of the last glacial period as is suggested by the EBSPs (Fig. 2) and the values of τ. Thus, the earlier larger range changes represented in the population genetic data would not be reflected in the paleodistributions estimated here.

### Validity of molecular clock-based methods

Although our inferences about changes in population size do not depend on knowledge about the absolute rates of sequence evolution, our capacity to identify the age of these patterns does assume that sequence evolution occurs in a roughly clocklike manner. Methods based on molecular clocks are inherently dependent on a number of assumptions, including the validity of underlying calibrations and the relative constancy of substitution rates over evolutionary time. Several high-profile studies published in recent years have raised some doubt as to whether sequence evolution typically occurs at a constant rate over time. Some analyses have suggested that the slow rate at which mildly deleterious mutations are eliminated through purifying selection leads to an apparent rate acceleration towards the present, or “time dependency” [105]–[107]. In addition, previous molecular-clock based studies of yuccas and yucca moths have revealed significant discrepancies between the ages of the two groups [51], [59], which may call into question the validity of the underlying calibrations.

Despite these caveats, we have confidence in the age estimates presented here. First, we are skeptical of the reports of ‘time dependency’ in molecular clocks; analytical work has shown that effective population sizes must be unrealistically large for purifying selection to produce the time dependency effect observed in empirical data [108], and a combination of simulation and empirical work has shown that the ‘time dependency’ seen in ancient DNA data is attributable to sampling artifacts [109]. Second, our clock estimates have been confirmed by independent analyses; the underlying molecular clock for the cpDNA data has previously been used to produce age estimates for the genus *Yucca* that are consistent with independent fossil data [59], and molecular clock studies examining rates of evolution in COI across the Insecta –including yucca moths– have recovered similar mutation rate estimates to those employed here [110]. Third, the age estimates in the moths rely on the same genes using a common fossil calibration point, and the likelihood ratio tests could not reject a molecular clock for these data. Thus, even if the data could not precisely estimate the *absolute* timing of demographic changes, they would provide reliable information about the *relative* timing of demographic changes. Our conclusion that the inferred population size changes were broadly contemporaneous within the moths should therefore be robust to any errors in our estimates of the substitution rates.

### Comparative phylogeography and the stability of ecological communities over time

Many studies have sought to determine whether co-distributed organisms show common phylogeographic patterns. The general expectation has been that to the extent that organisms experienced common geological and climactic changes, they can be expected to show congruent phylogeographic patterns. However, relatively few have identified shared histories across taxa, perhaps suggesting that species that co-occur today may not necessarily have responded to geohistorical events in a concerted fashion. The palaeorecord suggests that many species that currently co-occur have come into sympatry only recently, and many communities present at the last glacial maximum (LGM) are without modern analogues [43], [95], [111], [112]. Consequently, many ecologists have concluded that ecological communities are more individualistic (sensu Gleason [113]) than holistic (sensu Clements [114]).

Whether ecological communities are integrated wholes, or disconnected assortments of non-interacting species, was a highly contentious argument during the twentieth century [115], but by the 1970's and ‘80’s, however, accumulating palaeoecological data [116] and gradient analyses had largely settled the debate in favor of the individualistic, Gleasonian view. However, this consensus is at odds with the growing evidence that coevolutionary interactions shape much of the Earth's ecological and evolutionary processes [117]–[125]. A number of authors have observed that the individualistic view of ecological communities ignores the ubiquity of strong species interactions, whether mutualistic or antagonistic [21], [23], [24], [115]. Our results indicate that the highly specialized community of the Joshua tree and its associated yucca moths has shared a common biogeographic history over time. Thus, it seems that this community might be more Clementsian, and less Gleasonian.

It is certainly possible that climate changes might produce congruent phylogeographic and demographic changes in co-distributed organisms even in the absence of strong ecological interactions between them, and indeed this result has been identified in several empirical studies [3], [5], [24]. We have argued, however, congruent phylogeographic patterns will be more common among groups of organisms involved in obligate and highly specialized interactions. Our results are consistent with this hypothesis, but a complete evaluation of the frequency with which phylogeographic congruence occurs in different communities will require a much larger comparative study.

### Are yuccas exceptional?

Obligate pollination mutualisms are extremely specialized, and so it could be argued that communities like yuccas and yucca moths are not representative of most species assemblages. However, we maintain that the concerted demographic changes we see here are probably typical of many communities that contain highly specialized organisms, and that specialization is common not only in plants and insects, but in many groups that together account for much of the diversity of life. Herbivorous insects, for example, account for 26% of all described species [126], and it is increasingly clear that within these groups specialization is the norm [117], [121], [125], [127]–[132]. Recent work using DNA bar-coding to identify cryptic species has shown that extreme specificity is also typical within other hyper-diverse groups, such as parasitic wasps and flies [133], [134] and trematode worms [135], and may be more common than was previously supposed among ectomycorrhizal fungi [136], [137]. Much of the world may therefore be more Clementsian than Gleasonian.

A reasonable next step in addressing these questions would be to examine the biogeographic history of a greater diversity of herbivorous insects associated with this and other plants to determine the generality of our findings. If, as we argue, strong interactions between plants make these communities make these communities more likely to respond in a concerted fashion to climate change, then we should be able to identify many other communities that display similar patterns of common demographic and range changes over time.

Testing this prediction directly using palaeorecords may be challenging. Signatures of interactions between species are rarely preserved in the fossil record (but see [138], [139]). Similarly, many of the plant and insect species typically preserved in the palaeorecord are generalist species, such as wind-pollinated plants (conifers, oaks, grasses, sedges, and ragweeds) that do not form strong interactions with pollinators, and non-phytophagous insects, such as predaceous ground beetles and detritivores. However, combining fossil data with tools from statistical phylogeography and palaeodistribution modeling, as we have done here, can offer remarkable synergies and new insights into the history of ecological communities over time [82], [140]–[142]. This new synthesis in historical biogeography has already reshaped our understanding of the role of climate change in ecological and evolutionary processes, and may also enable improved predictions for how anthropogenic climate change will shape ecosystems over the coming century.

## Supporting Information

Figure S1 **A comparison of non-analogous climates between the present day and the LGM.** **D**ark cells represent locations where at least one climatic variable was more extreme during the LGM than any climate currently present within 500 km of the range of *Y. brevifolia*.
(TIF)

Figure S2 **Extended Bayesian Skyline Plots inferred using BEAST v. 1.5.3 from sequence data simulated for each species under four alternative demographic scenarios.** The graphs depict mean values, averaged across one hundred (100) separate data sets. Note that the y-axes are not to scale.
(TIF)

Table S1 Collection localities for samples included in this study.
(PDF)

Table S2 Collection localities and GenBank accession numbers for insect samples.
(PDF)

Table S3 Collection localities and GenBank accession numbers for plant tissue.
(PDF)

Table S4 Worldclim variables included in estimation of paleodistributions.
(PDF)

Table S5 Location, carbon-14 ages, and calendar year ages estimated using CALIB 5.0.2 of fossil records for *Y. brevifolia*..
(PDF)

Table S6 Summary of Lamarc simulations.
(PDF)

File S1 Google Earth animation of continuous phylogeographic analysis.
(KML)

## References

[1] Arbogast BS, Kenagy GJ (2001). Comparative phylogeography as an integrative approach to historical biogeography.. Journal of Biogeography.

[2] Avise JC (2009). Phylogeography: retrospect and prospect.. Journal of Biogeography.

[3] Hewitt GM (1999). Post-glacial re-colonization of European biota.. Biological Journal of the Linnean Society.

[4] Soltis DE, Morris AB, McLachlan JS, Manos PS, Soltis PS (2006). Comparative phylogeography of unglaciated eastern North America.. Molecular Ecology.

[5] Lapointe FJ, Rissler LJ (2005). Congruence, Consensus, and the Comparative Phylogeography of Codistributed Species in California.. The American Naturalist.

[6] Hewitt GM (1996). Some genetic consequences of ice ages, and their role in divergence and speciation.. Biological Journal of the Linnean Society.

[7] Santucci F, Emerson BC, Hewitt GM (1998). Mitochondrial DNA phylogeography of European hedgehogs.. Molecular Ecology.

[8] Dumolin-Lapegue S, Demesure B, Fineschi S, Corre VL, Petit RJ (1997). Phylogeographic Structure of White Oaks Throughout the European Continent.. Genetics.

[9] Hewitt G (2000). The genetic legacy of the Quaternary ice ages.. Nature.

[10] Moussalli A, Moritz C, Williams SE, Carnaval AC (2009). Variable responses of skinks to a common history of rainforest fluctuation: concordance between phylogeography and palaeo-distribution models.. Molecular Ecology.

[11] Carstens B, Brunsfeld SJ, Demboski JR, Good J, Sullivan J (2005). Investigating the evolutionary history of the Pacific Northwest mesic forest ecosystem: hypothesis testing within a comparative phylogeographic framework.. Evolution.

[12] Sunnucks P, Blacket MJ, Taylor JM, Sands CJ, Ciavaglia SA (2006). A tale of two flatties: different responses of two terrestrial flatworms to past environmental climatic fluctuations at Tallaganda in montane southeastern Australia.. Molecular Ecology.

[13] Solomon SE, Bacci M, Martins J, Vinha GGa, Mueller UG (2008). Paleodistributions and comparative molecular phylogeography of leafcutter ants (*Atta* spp.) provide new insight into the origins of Amazonian diversity.. PLoS ONE.

[14] Zink RM, Kessen AE, Line TV, Blackwell-Rago RC (2001). Comparative phylogeography of some aridland bird species.. The Condor.

[15] Mikheyev AS, Tanya V, Ulrich GM (2008). Phylogeography of post-Pleistocene population expansion in a fungus-gardening ant and its microbial mutualists.. Molecular Ecology.

[16] Wares JP, Cunningham CW (2001). Phylogeography and historical ecology of the North Atlantic intertidal.. Evolution.

[17] Carstens BC, Richards CL (2007). Integrating coalescent and ecological niche modeling in comparative phylogeography.. Evolution.

[18] Carstens B, Stevenson A, Degenhardt J, Sullivan J (2004). Testing nested phylogenetic and phylogeographic hypotheses in the *Plethodon vandykei* species group.. Systematic Biology.

[19] Carstens BC, Degenhardt JD, Stevenson AL, Sullivan J (2005). Accounting for coalescent stochasticity in testing phylogeographical hypotheses: modelling Pleistocene population structure in the Idaho giant salamander *Dicamptodon aterrimus*.. Molecular Ecology.

[20] Taberlet P, Fumagalli L, WustSaucy AG, Cosson JF (1998). Comparative phylogeography and postglacial colonization routes in Europe.. Molecular Ecology.

[21] Jackson ST, Overpeck J (2000). Responses of plant populations and communities to environmental changes of the late Quaternary.. Paleobiology.

[22] Whiteman NK, Kimball RT, Parker PG (2007). Co-phylogeography and comparative population genetics of the threatened Galápagos hawk and three ectoparasite species: ecology shapes population histories within parasite communities.. Molecular Ecology.

[23] Callaway RM (1997). Positive interactions in plant communities and the individualistic-continuum concept.. Oecologia.

[24] Rowe KC, Heske EJ, Paige KN (2006). Comparative phylogeography of eastern chipmunks and white-footed mice in relation to the individualistic nature of species.. Molecular Ecology.

[25] Pellmyr O (2003). Yuccas, yucca moths and coevolution: a review.. Annals of the Missouri Botanical Garden.

[26] Weiblen GD (2002). How to be a fig wasp.. Annual Review of Entomology.

[27] Janzen D (1979). How to be a fig.. Annual Review of Ecology and Systematics.

[28] Herre EA, Jandér KC, Machado CA (2008). Evolutionary Ecology of Figs and Their Associates: Recent Progress and Outstanding Puzzles.. Annual Review of Ecology, Evolution, and Systematics.

[29] Haine E, Martin J, Cook J (2006). Deep mtDNA divergences indicate cryptic species in a fig-pollinating wasp.. BMC Evolutionary Biology.

[30] Carlos L-V, Dale JD, James MC, Jean-Yves R (2002). Revision of the Australian species of *Pleistodontes* (Hymenoptera: Agaonidae) fig-pollinating wasps and their host-plant associations.. Zoological Journal of the Linnean Society.

[31] Machado CA, Robbins N, Gilbert MTP, Herre EA (2005). Critical review of host specificity and its coevolutionary implications in the fig/fig-wasp mutualism.. Proceedings of the National Academy of Sciences.

[32] Molbo D, Machado CA, Sevenster JG, Keller L, Herre EA (2003). Cryptic species of fig-pollinating wasps: implications for the evolution of the fig-wasp mutualism, sex allocation, and precision of adaptation.. Proceedings of the National Academy of Sciences.

[33] Pellmyr O, Balcazar-Lara M, Segraves KA, Althoff DM, Littlefield RJ (2008). Phylogeny of the pollinating yucca moths, with revision of Mexican species (*Tegeticula* and *Parategeticula*; Lepidoptera, Prodoxidae).. Zoological Journal of the Linnean Society.

[34] Pellmyr O (1999). Systematic revision of the yucca moths in the *Tegeticula yuccasella* complex (Lepidoptera : Prodoxidae) north of Mexico.. Systematic Entomology.

[35] Pellmyr O, Balcazar Lara M (2000). Systematics of the Yucca Moth genus *Parategeticula* (Lepidoptera : Prodoxidae), with description of three Mexican species.. Annals of the Entomological Society of America.

[36] Pellmyr O, Leebens-Mack J (2000). Reversal of mutualism as a mechanism for adaptive radiation in yucca moths.. American Naturalist.

[37] Pellmyr O, Leebens-Mack J, Huth CJ (1996). Non-mutualistic yucca moths and their evolutionary consequences.. Nature.

[38] Segraves KA, Pellmyr O (2004). Testing the ‘Out of Florida’ hypothesis on the origin of cheating in the yucca-yucca moth mutualism.. Evolution.

[39] Segraves KA, Althoff DM, Pellmyr O (2008). The evolutionary ecology of cheating: does superficial oviposition facilitate the evolution of a cheater yucca moth?. Ecological Entomology.

[40] West SA, Herre EA, Donald MW, Green PRS (1996). The ecology and evolution of the New World non-pollinating fig wasp communities.. Journal of Biogeography.

[41] Bronstein J (1991). The nonpollinating wasp fauna of *Ficus pertusa*: exploitation of a mutualism?. Oikos.

[42] Machado CA, Herre EA, McCafferty S, Bermingham E (1996). Molecular phylogenies of fig pollinating and non-pollinating wasps and the implications for the origin and evolution of the fig-fig wasp mutualism.. Journal of Biogeography.

[43] Van Devender TR, Betancourt JL, Van Devender TR, Martin PS (1990). Late quaternary vegetation and climate of the Sonoran desert, United States and Mexico.. Packrat Middens: The Last 40,000 Years of Biotic Change.

[44] Spaulding WG (1985). Vegetation and climates of the last 45,000 years in the vicinity of the Nevada Test Site, south-central Nevada. United States Geological Survey.. Paper.

[45] Cole K, Ironside K, Eischeid J, Garfin G, Duffy P (2011). Past and ongoing shifts in Joshua tree support future modeled range contraction.. Ecological Applications.

[46] Anderson RS, Van Devender TR (1991). Comparison of pollen and macrofossils in packrat (*Neotoma*) middens: A chronological sequence from the Waterman Mountains of southern Arizona, U.S.A.. Review of Palaeobotany and Palynology.

[47] Koehler PA, Anderson RS (1995). Thirty thousand years of vegetation changes in the Alabama Hills, Owens Valley, California.. Quarternary Research.

[48] Van Devender T (1987). Holocene vegetation and climate in the Puerto Blanco Mountains, Southwestern Arizona.. Quaternary Research.

[49] Cole KL, Ironside K, Eischeid J, Garfin G, Duffy PB (2010). Past and ongoing shifts in Joshua tree distribution support future modeled range contraction.. Ecological Applications.

[50] Pellmyr O, Segraves KA (2003). Pollinator divergence within an obligate mutualism: two yucca moth species (Lepidoptera; Prodoxidae: *Tegeticula*) on the Joshua Tree (*Yucca brevifolia*; Agavaceae).. Annals of the Entomological Society of America.

[51] Smith CI, Godsoe WKW, Tank S, Yoder JB, Pellmyr O (2008). Distinguishing coevolution from covicariance in an obligate pollination mutualism: Asynchronous divergence in Joshua tree and its pollinators.. Evolution.

[52] Pellmyr O, Balcazar-Lara M, Althoff DM, Segraves KA, Leebens Mack JH (2005). Phylogeny and life history evolution of *Prodoxus* yucca moths (Lepidoptera: Prodoxidae).. Systematic Entomology.

[53] Gillson L, Ekblom A, Willis K, Froyd C (2008). Holocene palaeo-invasions: the link between pattern, process and scale in invasion ecology?. Landscape Ecology.

[54] Moorcroft PR, Pacala SW, Lewis MA (2006). Potential role of natural enemies during tree range expansions following climate change.. Journal of Theoretical Biology.

[55] Edgar R (2004). MUSCLE: a multiple sequence alignment method with reduced time and space complexity.. BMC Bioinformatics.

[56] Stephens M, Smith NJ, Donnelly P (2001). A new statistical method for haplotype reconstruction from population data.. American Journal of Human Genetics.

[57] Posada D, Crandall KA (1998). Modeltest: testing the model of DNA substitution.. Bioinformatics.

[58] Posada D, Crandall KA (2001). Selecting the best-fit model of nucleotide substitution.. Systematic Biology.

[59] Smith CI, Pellmyr O, Althoff DM, Balcázar-Lara M, Leebens Mack JH (2008). Pattern and timing of diversification in *Yucca* (Agavaceae): specialized pollination does not escalate rates of diversification.. Proceedings of the Royal Society of London Series B.

[60] Mcdonald JH, Kreitman M (1991). Adaptive protein evolution at the AdH lous in *Drosophila*.. Nature.

[61] Rozas J, Sanchez-DelBarrio JC, Messeguer X, Rozas R (2003). DnaSP, DNA polymorphism analyses by the coalescent and other methods.. Bioinformatics.

[62] Excoffier L, Smouse P, Quattro J (1992). Analysis of molecular variance inferred from metric distance among DNA haplotypes: Application to human mitochondrial DNA restriction data.. Genetics.

[63] Kuhner MK (2006). LAMARC 2.0: maximum likelihood and Bayesian estimation of population parameters.. Bioinformatics.

[64] Drummond A, Rambaut A (2007). BEAST: Bayesian evolutionary analysis by sampling trees.. BMC Evolutionary Biology.

[65] Heled J, Drummond A (2008). Bayesian inference of population size history from multiple loci.. BMC Evolutionary Biology.

[66] Fu YX (1997). Statistical tests of neutrality of mutations against population growth, hitchhiking and background selection.. Genetics.

[67] Ramos-Onsins SE, Rozas J (2002). Statistical Properties of New Neutrality Tests Against Population Growth.. Molecular Biology and Evolution.

[68] Rogers AR, Harpending H (1992). Population growth makes waves in the distribution of pairwise genetic differences.. Molecular Biology and Evolution.

[69] Pannell JR (2003). Coalescence in a metapopulation with recurrent local extinction and recolonization.. Evolution.

[70] Smouse PE, Long JC, Sokal RR (1986). Multiple Regression and Correlation Extensions of the Mantel Test of Matrix Correspondence.. Systematic Zoology.

[71] Excoffier L, Lischer HEL (2010). Arlequin suite ver 3.5: A new series of programs to perform population genetics analyses under Linux and Windows.. Molecular Ecology Resources.

[72] Ersts PJ (2007). Geographic Distance Matrix Generator..

[73] Wright S (1978). Evolution and the Genetics of Populations Vol 4: Variability within and among Natural Populations.

[74] Lemey P, Rambaut A, Welch JJ, Suchard MA (2010). Phylogeography Takes a Relaxed Random Walk in Continuous Space and Time.. Molecular Biology and Evolution.

[75] Bielejec F, Rambaut A, Suchard MA, Lemey P (2011). SPREAD: Spatial Phylogenetic Reconstruction of Evolutionary Dynamics.. Bioinformatics.

[76] Merriam CH, Merriam CH (1893). Notes on the geographical and vertical distributions of cactuses, yuccas, and agaves in the deserts and desert ranges of southern California, southern Nevada, northwestern Arizona and southwestern Utah.. The Death Valley Expedition.

[77] Rowlands PG (1978). The vegetation dynamics of the Joshua Tree (*Yucca brevifolia* Engelm.) in the southwestern United States of America [PhD Dissertation].

[78] McKelvey SD (1938). Yuccas of the Southwestern United States.

[79] Cole KL, Pohs K, Cannella JA (2003). Digital range map of Joshua tree (*Yucca brevifolia*).. http://sbsc.wr.usgs.gov/cprs/research/projects/global_change/RangeMaps/YuccaBrevifoliaDistributionMap.pdf.

[80] Stuiver M, Reimer P (1993). Extended 14C database and revised CALIB radiocarbon calibration program.. Radiocarbon.

[81] Van Devender TR, Martin PS (1990). Late quaternary vegetation and climate of the Chihuahuan desert, United States and Mexico.. Packrat Middens: The Last 40,000 Years of Biotic Change.

[82] Richards CL, Carstens BC, Knowles LL (2007). Distribution modelling and statistical phylogeography: an integrative framework for generating and testing alternative biogeographical hypotheses.. Journal of Biogeography.

[83] Kutzbach JE, Guetter PJ (1986). The influence of changing orbital parameters and surface boundary conditions on climate simulations for the past 18,000 years.. Journal of the Atmospheric Sciences.

[84] Elith J, Graham CH, Anderson RP, Dudik M, Ferrier S (2006). Novel methods improve prediction of species' distributions from occurrence data.. Ecography.

[85] R Development Core Team (2006). R: A language and environment for statistical computing.

[86] Elith J, Lethwick JR, Hastie T (2008). A working guide to boosted regression trees.. Journal of Animal Ecology.

[87] Ridgeway G (2006). GBM: Generalized Boosted Regression Models.. http://www.i-pensieri.com/gregr/gbm.shtml.

[88] Wright HE, Kutzbach JE, Webb T, Ruddiman WF, Street-Perrott FA (1993). Global Climate Sicne the Last Glacial Maximum.

[89] Nogués-Bravo D (2009). Predicting the past distribution of species climate niches.. Global Ecology and Biogeography.

[90] Godsoe W (2009). Regional Variation Exaggerates Ecological Divergence in Niche Models.. Systematic Biology.

[91] Freeman E (2007). PresenceAbsence: An R package for presence-absence model evaluation.. http://cran.r-project.org/web/packages/PresenceAbsence/index.html.

[92] Liu C, Berry PM, Dawson TP, Pearson RG (2005). Selecting thresholds of occurrence in the prediction of species distributions.. Ecography.

[93] Kuhner M, Yamamato J, Felsenstein J (1998). Maximum likelihood estimation of population growth rates based on the coalescent.. Genetics.

[94] Maddison WP, Maddison DR (2006). Mesquite: A modular system for evolutionary analysis.. http://mesquiteproject.org/mesquite/mesquite.html.

[95] Betancourt J, Betancourt J, Van Devender TR, Martin PS (1990). Late Quaternary biogeography of the Colorado Plateau.. Packrat middens: The last 40,000 years of biotic change.

[96] Vilella AJ, Blanco-Garcia A, Hutter S, Rozas J (2005). VariScan: Analysis of evolutionary patterns from large-scale DNA sequence polymorphism data.. Bioinformatics.

[97] Landis RJ, Koch GG (1977). The measurement of observer agreement for categorical data.. Biometrics.

[98] Fay JC, Wu CI (1999). A human population bottleneck can account for the discordance between patterns of mitochondrial versus nuclear DNA variation.. Molecular Biology and Evolution.

[99] Drummond CS, Xue HJ, Yoder JB, Pellmyr O (2010). Host-associated divergence and incipient speciation in the yucca moth *Prodoxus coloradensis* (Lepidoptera: Prodoxidae) on three species of host plants.. Heredity.

[100] Vander Wall SB, Esque TC, Garnett M, Waitman B (2006). Joshua tree (*Yucca brevifolia*) seed are dispersed by seed-caching rodents.. Ecoscience.

[101] Hewitt GM (1996). Some genetic consequences of ices ages, and their role in divergence and speciation.. Biological Journal of the Linnean Society.

[102] Tajima F (1989). The Effect of Change in Population Size on DNA Polymorphism.. Genetics.

[103] Bazin E, Glemin S, Galtier N (2006). Population Size Does Not Influence Mitochondrial Genetic Diversity in Animals.. Science.

[104] Nielsen R, Bustamante C, Clark AG, Glanowski S, Sackton TB (2005). A Scan for Positively Selected Genes in the Genomes of Humans and Chimpanzees.. PLoS Biology.

[105] Ho SYW, Larson G (2006). Molecular clocks: when times are a-changin'.. Trends in Genetics.

[106] Ho SYW, Phillips MJ, Cooper A, Drummond AJ (2005). Time dependency of molecular rate estimates and systematic overestimation of recent divergence times.. Molecular Biology and Evolution.

[107] Ho SYW, Shapiro B, Phillips MJ, Cooper A, Drummond AJ (2007). Evidence for Time Dependency of Molecular Rate Estimates.. Systematic Biology.

[108] Woodhams M (2006). Can Deleterious Mutations Explain the Time Dependency of Molecular Rate Estimates?. Molecular Biology and Evolution.

[109] Debruyne R, Poinar HN (2009). Time Dependency of Molecular Rates in Ancient DNA Data Sets, A Sampling Artifact?. Systematic Biology.

[110] Gaunt MW, Miles MA (2002). An insect molecular clock dates the origin of the insects and accords with palaeontological and biogeographic landmarks.. Molecular Biology and Evolution.

[111] Thompson RS, Anderson KH (2000). Biomes of western North American at 18,000, 6000, and 0 14C yr BP reconstructed from pollen and packrat midden data.. Journal of Biogeography.

[112] Jackson ST, Williams JW (2004). Modern analogs in quaternary paleoecology: Here today, gone yesterday, gone tomorrow?. Annual Review of Earth and Planetary Sciences.

[113] Gleason HA (1926). The Individualistic Concept of the Plant Association.. Bulletin of the Torrey Botanical Club.

[114] Clements FE (1916). Plant Succession An Analysis of the Development of Vegetation.

[115] Odenbaugh J (2007). Seeing the Forest and the Trees: Realism about Communities and Ecosystems.. Philosophy of Science.

[116] Davis MB, Wright HE (1983). Holocene vegetational history of the eastern United States.. Late Quaternary Environments of the United States.

[117] Sargent RD (2004). Floral symmetry affects speciation rates in angiosperms.. Proceedings of the Royal Society of London Series B.

[118] Farrell BD, Dussourd DE, Mitter C (1991). Escalation of plant defense: Do latex and resin canals spur plant diversification?. The American Naturalist.

[119] Thompson JN (2005). The Geographic Mosaic of Coevolution.

[120] Farrell BD (1998). Inordinate fondness explained: why are there so many beetles?. Science.

[121] Ehrlich P, Raven P (1964). Butterflies and plants: a study in coevolution.. Evolution.

[122] Grant V (1949). Pollination systems as isolating mechanisms in angiosperms.. Evolution.

[123] Mitter C, Farrell B, Wiegmann B (1988). The phylogenetic study of adaptive zones: has phytophagy promoted insect diversification?. American Naturalist.

[124] Stebbins GL (1970). Adaptive radiation of reproductive characteristics in angiosperms, I: Pollination mechanisms.. Annual Review of Ecology and Systematics.

[125] Armbruster W, Muchhala N (2009). Associations between floral specialization and species diversity: cause, effect, or correlation?. Evolutionary Ecology.

[126] Strong DR, Lawton JH, Southwood SR (1984). Insects on Plants.

[127] Jaenike J (1990). Host specialization in phytophagous insects.. Annual Review of Ecology and Systematics.

[128] Futuyma DJ, Moreno G (1988). The evolution of ecological specialization.. Annual Review of Ecology and Systematics.

[129] Wahlberg N (2001). The phylogenetics and biochemistry of host-plant specialization in Melitaeine butterflies (Lepidoptera : Nymphalidae).. Evolution.

[130] Dyer LA, Singer MS, Lill JT, Stireman JO, Gentry GL (2007). Host specificity of Lepidoptera in tropical and temperate forests.. Nature.

[131] Bickford D, Lohman DJ, Sodhi NS, Ng PKL, Meier R (2007). Cryptic species as a window on diversity and conservation.. Trends in Ecology & Evolution.

[132] Hebert PDN, Penton EH, Burns JM, Janzen DH, Hallwachs W (2004). Ten species in one: DNA barcoding reveals cryptic species in the neotropical skipper butterfly *Astraptes fulgerator*.. Proceedings of the National Academy of Sciences of the United States of America.

[133] Smith MA, Rodriguez JJ, Whitfield JB, Deans AR, Janzen DH (2008). Extreme diversity of tropical parasitoid wasps exposed by iterative integration of natural history, DNA barcoding, morphology, and collections.. Proceedings of the National Academy of Sciences.

[134] Smith MA, Woodley NE, Janzen DH, Hallwachs W, Hebert PDN (2006). DNA barcodes reveal cryptic host-specificity within the presumed polyphagous members of a genus of parasitoid flies (Diptera: Tachinidae).. Proceedings of the National Academy of Sciences of the United States of America.

[135] Locke SA, Mclaughlin DJ, Marcogliese DJ (2010). DNA barcodes show cryptic diversity and a potential physiological basis for host specificity among *Diplostomoidea* (Platyhelminthes: Digenea) parasitizing freshwater fishes in the St. Lawrence River, Canada.. Molecular Ecology.

[136] Leho T, Triin S, Teele J, Ivika O, Sergei P (2009). Revisiting ectomycorrhizal fungi of the genus *Alnus*: differential host specificity, diversity and determinants of the fungal community.. New Phytologist.

[137] Leho T, Teele J, Bryony MH, Kessy A, Triin S (2008). Strong host preference of ectomycorrhizal fungi in a Tasmanian wet sclerophyll forest as revealed by DNA barcoding and taxon-specific primers.. New Phytologist.

[138] Labandeira CC, Dilcher DL, Davis DR, Wagner DL (1994). Ninety-seven million years of angiosperm-insect association: paleobiological insights into the meaning of coevolution.. Proceedings of the National Academy of Sciences of the United States of America.

[139] Wilf P, Labandeira CC (1999). Response of Plant-Insect Associations to Paleocene-Eocene Warming.. Science.

[140] Knowles LL, Carstens BC, Keat ML (2007). Coupled Genetic and Ecological-Niche Models for Examining How Past Species Distributions Contribute to Population Divergence.. Current Biology.

[141] Wiens JJ, Donoghue MJ (2004). Historical biogeography, ecology, and species richness.. Trends in Ecology and Evolution.

[142] Knowles L (2004). The burgeoning field of statistical phylogeography.. Journal of Evolutionary Biology.

