# Supplementary figures and images for: Comparative Phylogeography of a Coevolved Community: Concerted Population Expansions in Joshua Trees and Four Yucca Moths
Source: PLoS One. 2011 Oct 18;6(10):e25628. doi: 10.1371/journal.pone.0025628 (PMC3196504; doi:10.1371/journal.pone.0025628)

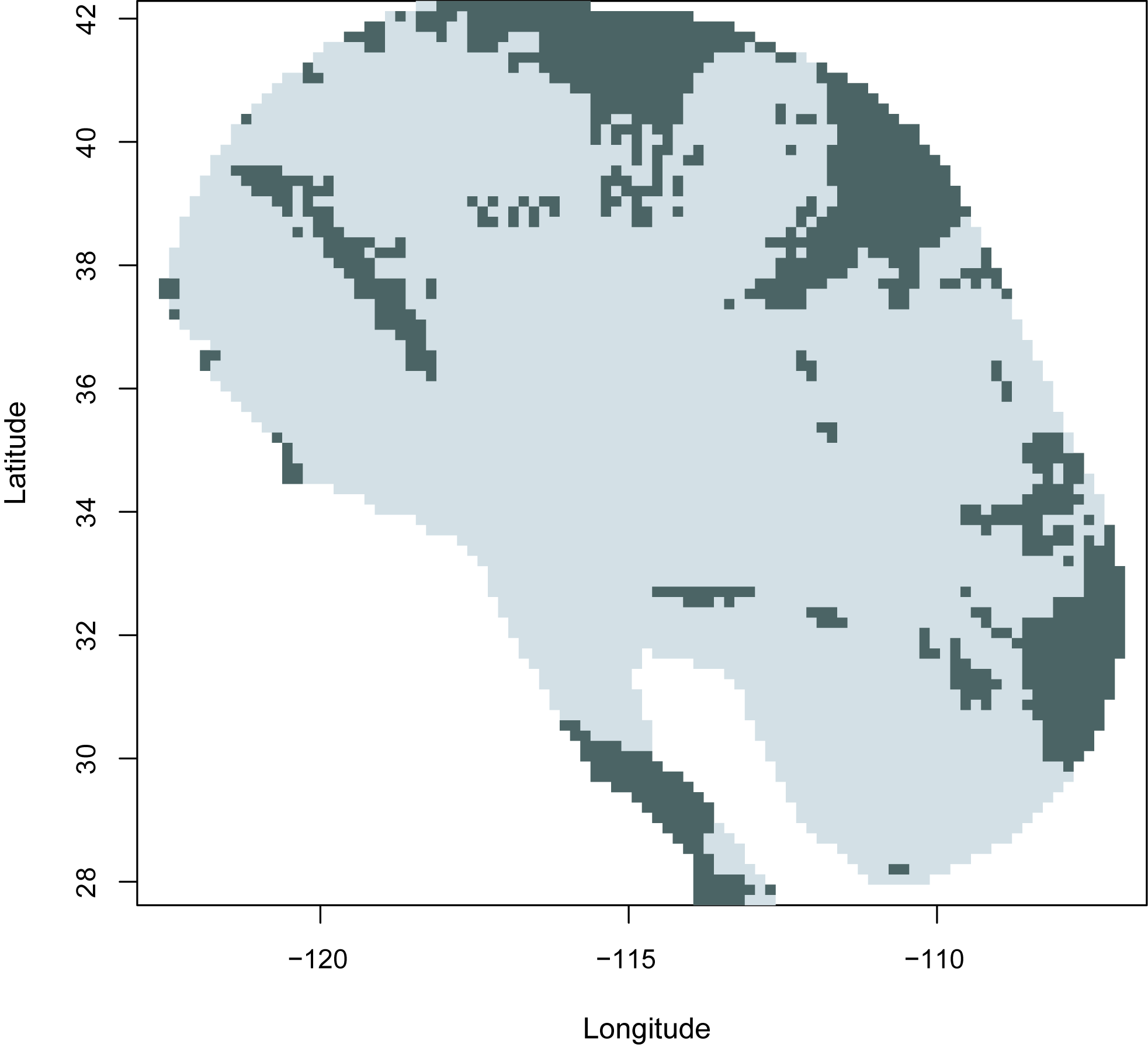

Supplement: Figure S1 — A comparison of non-analogous climates between the present day and the LGM. Dark cells represent locations where at least one climatic variable was more extreme during the LGM than any climate currently present within 500 km of the range of Y. brevifolia. (TIF) [file pone.0025628.s001.tif]

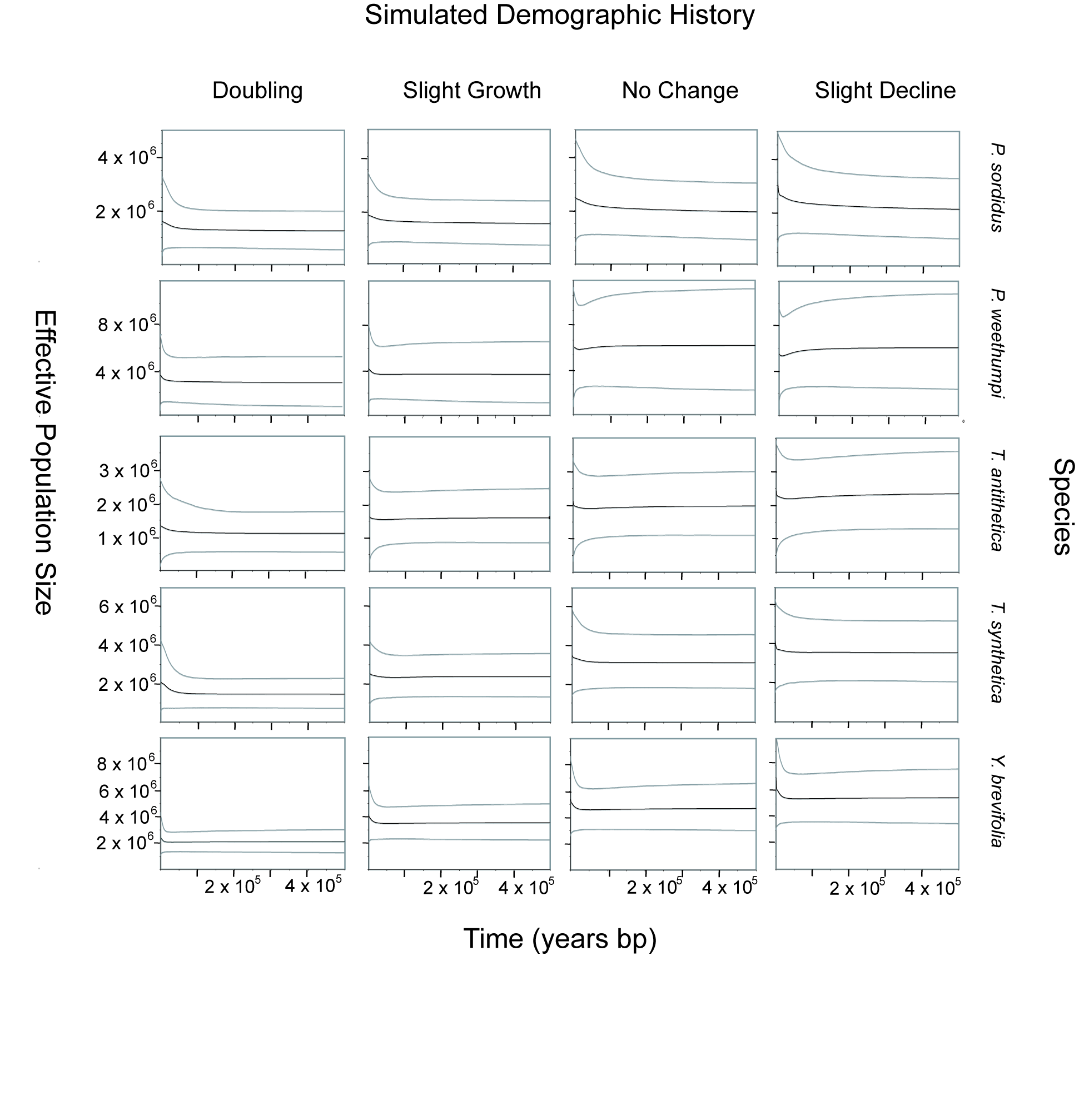

Supplement: Figure S2 — Extended Bayesian Skyline Plots inferred using BEAST v. 1.5.3 from sequence data simulated for each species under four alternative demographic scenarios. The graphs depict mean values, averaged across one hundred (100) separate data sets. Note that the y-axes are not to scale. (TIF) [file pone.0025628.s002.tif]
