# Supplementary material for: Comparative Phylogeography of a Coevolved Community: Concerted Population Expansions in Joshua Trees and Four Yucca Moths
Source: PLoS One. 2011 Oct 18;6(10):e25628. doi: 10.1371/journal.pone.0025628 (PMC3196504; doi:10.1371/journal.pone.0025628)
Supplement: Table S1 — Collection localities for samples included in this study. (PDF) [file pone.0025628.s003.pdf]

# Supporting Information

## Table S1: Collection Localities For Samples Included in This Study

| Site # | Site Name | Longitude | Latitude |
|---|---|---|---|
| 1 | Joshua Tree N.P. | -116.2221 | 34.0561 |
| 2 | Palmdale, CA | -117.8077 | 34.4756 |
| 3 | Lancaster, CA | -118.5038 | 34.7732 |
| 4 | Kramer Hills | -117.5012 | 34.9122 |
| 5 | Fossil Bed Road | -117.0057 | 34.9874 |
| 6 | Freemont  Mts | -117.4989 | 35.2226 |
| 7 | Trona Road | -117.5871 | 35.474 |
| 8 | Walker Pass | -118.005367 | 35.6507 |
| 9 | Cactus Flats | -117.9351 | 36.21866 |
| 10 | Tin Mountain | -117.5032 | 36.8599 |
| 11 | Eureka Valley | -117.8273 | 37.3794 |
| 12 | Clayton Valley | -117.733333 | 37.583333 |
| 13 | Montezuma Peak | -117.2722 | 37.7486 |
| 14 | Gold Point | -117.375 | 37.3588 |
| 15 | Bonnie Claire Flat | -117.2565 | 37.0691 |
| 16 | Crater Valley | -116.579167 | 36.820483 |
| 17 | Nevada Test Site I | -116.065267 | 36.959067 |
| 18 | Nevada Test Site II | -115.989483 | 36.979433 |
| 19 | Tikaboo Valley | -115.506 | 37.4833 |
| 20 | Sheep Range | -115.18515 | 37.1783 |
| 21 | Eightmile Valley | -115.030383 | 37.374017 |
| 22 | Delmar Road | -114.815817 | 37.524917 |
| 23 | Sheep Pass | -115.298783 | 36.829217 |
| 24 | Sawmill Road | -115.057717 | 36.697167 |
| 25 | Yucca Forest | -115.057717 | 36.478183 |
| 26 | Dry Lake | -114.9571 | 36.5127 |
| 27 | Shivwits | -113.8969 | 37.0641 |
| 28 | Indian Ridge | -115.6897 | 36.500667 |
| 29 | Kyle Canyon | -115.5155 | 36.2662 |
| 30 | Lovell Canyon | -115.553 | 36.0478 |
| 31 | Jean, NV | -115.3777 | 35.8053 |
| 32 | Searchlight | -115.1391 | 35.5138 |
| 33 | Cima | -115.6255 | 35.3961 |
| 34 | Kingston  Mountains | -115.8356 | 35.7703 |
| 35 | Avawatz  Mtns | -116.348283 | 35.57027 |
| 36 | Dolan Springs | -114.2608 | 35.5979 |
| 37 | Alamo Crossing | -114.073833 | 34.806183 |
| 38 | Alamo Lake | -113.3932 | 34.1998 |
| 39 | Joshua Tree Parkway | -113.0699 | 34.2268 |
