# Supplementary material for: Comparative Phylogeography of a Coevolved Community: Concerted Population Expansions in Joshua Trees and Four Yucca Moths
Source: PLoS One. 2011 Oct 18;6(10):e25628. doi: 10.1371/journal.pone.0025628 (PMC3196504; doi:10.1371/journal.pone.0025628)
Supplement: Table S2 — Collection localities and GenBank accession numbers for insect samples. (PDF) [file pone.0025628.s004.pdf]

Table S2: Collection Localities and GenBank Accession Numbers for Insect Samples

Taxon	Site #	GenBank Accession COI	GenBank Accession ND5	GenBank Accession EF1a
P. sordidus	2	GU374139 - GU374143	GU374195- GU374199	GU374319 - GU374330
P. sordidus	13	GU374138 GU374149- GU374151	GU374194, GU374205- GU374207	GU374311-GU374312, GU374343- GU374348
P. sordidus	27	GU374127 - GU374132	GU374183	GU374295- GU374306
P. sordidus	33	GU374144- GU374148	GU374200- GU374204	GU374331- GU374342
P. sordidus	37	GU374133 - GU374137	GU374189 -GU374193	GU374313 - GU374310
P. weethumpi	2	GU374158- GU374159, GU374176- GU374181	GU374214- GU374215, GU374230- GU374235	GU374251-GU374254, GU374281- GU374290
P. weethumpi	13	GU374171- GU374175	GU374225- GU374229	GU374271-GU374280
P. weethumpi	27	GU374154- GU374157	GU374210- GU374213	GU374243- GU374250
P. weethumpi	33	GU374164- GU374170, GU374182	GU374219- GU374224, GU374236	GU374259-GU374268, GU374291-GU374294
P. weethumpi	37	GU374152- GU374153, GU374160- GU374163	GU374208- GU374209, GU374216- GU374218	GU374237- GU374242, GU374255-GU374258
T. antithetica	19	EU585179- EU585184	EU584623- EU584627	EU584892, EU584893
T. antithetica	25	EU585157- EU585160	EU584601- EU584604	EU584859- EU584866
T. antithetica	27	EU585126- EU585135	EU584570- EU584579	EU584799-EU584812
T. antithetica	30	EU585147- EU585152	EU584591- EU584596	EU584847-EU584852
T. antithetica	33	EU585221	EU584665	EU584894, EU584895
T. antithetica	37	EU585137- EU585138, EU585141-EU585144	EU584581- EU584582, EU584585-EU584588	EU584815-EU584818, EU584823-EU584830
T. synthetica	2	EU585251- EU585254	EU584695- EU584698	EU584974- EU584983
T. synthetica	9	EU585273- EU585277	EU584717- EU584721	EU585004- EU585009
T. synthetica	11	EU585294- EU585299	EU584738- EU584743	EU585040- EU585045
T. synthetica	17	EU585309- EU585315	EU584753- EU584755, EU584758- EU584759	EU585046,EU585047, EU585050, EU585051
T. synthetica	19	EU585174, EU585177, EU585178, EU585180- EU585184	EU584621-EU584628	EU585052- EU585055, EU585068- EU585075
