# Supplementary material for: Comparative Phylogeography of a Coevolved Community: Concerted Population Expansions in Joshua Trees and Four Yucca Moths
Source: PLoS One. 2011 Oct 18;6(10):e25628. doi: 10.1371/journal.pone.0025628 (PMC3196504; doi:10.1371/journal.pone.0025628)
Supplement: Table S3 — Collection localities and GenBank accession numbers for plant tissue. (PDF) [file pone.0025628.s005.pdf]

Table S3: Collection Localities and GenBank Accession Numbers for Plant Tissue

Taxon	Site #	Site Name	GenBank Accession trnT-L	GenBank Accession trnL-trnF	GenBank Accession clpP intron 2
Y. brevifolia	1	Joshua Tree N.P.	EU585424	EU585501	EU585578
			EU585425	EU585502	EU585579
Y. brevifolia	2	Palmdale, CA	EU585422	EU585499	EU585576
			EU585423	EU585500	EU585577
Y. brevifolia	3	Lancaster, CA	EU585410	EU585487	EU585564
			EU585411	EU585488	EU585565
Y. brevifolia	4	Kramer Hills	EU585418	EU585495	EU585572
			EU585419	EU585496	EU585573
Y. brevifolia	5	Fossil Bed Road	EU585420	EU585497	EU585574
			EU585421	EU585498	EU585575
Y. brevifolia	6	Freemont Mts	EU585416	EU585493	EU585570
			EU585417	EU585494	EU585571
Y. brevifolia	7	Trona Road	EU585414	EU585491	EU585568
			EU585415	EU585492	EU585569
Y. brevifolia	8	Walker Pass	EU585412	EU585489	EU585566
			EU585413	EU585490	EU585567
Y. brevifolia	9	Cactus Flats	EU585398	EU585475	EU585552
			EU585399	EU585476	EU585553
Y. brevifolia	10	Tin Mountain	EU585400	EU585477	EU585554
			EU585401	EU585478	EU585555
Y. brevifolia	11	Eureka Valley	EU585396	EU585473	EU585550
			EU585397	EU585474	EU585551
Y. brevifolia	12	Clayton Valley	EU585391-	EU585468-	EU585545-
			EU585393	EU585470	EU585547
Y. brevifolia	13	Montezuma Peak	EU585387	EU585464	EU585541
			EU585388	EU585465	EU585542
Y. brevifolia	14	Gold Point	EU585394	EU585471	EU585548
			EU585395	EU585472	EU585549
Y. brevifolia	15	Bonnie Claire Flat	EU585402	EU585479	EU585556
			EU585403	EU585480	EU585557
Y. brevifolia	16	Crater Valley	EU585404	EU585481	EU585558
			EU585405	EU585482	EU585559
Y. brevifolia	17	Nevada Test Site I	EU585406	EU585483	EU585560
			EU585407	EU585484	EU585561
Y. brevifolia	18	Nevada Test Site II	EU585408	EU585485	EU585562
			EU585409	EU585486	EU585563
Y. brevifolia	19	Tikaboo Valley	EU585389	EU585466	EU585543
			EU585390	EU585467	EU585544
Y. brevifolia	20	Sheep Range	EU585371	EU585448	EU585525
			EU585372	EU585449	EU585526
Y. brevifolia	21	Eightmile Valley	EU585375	EU585452	EU585529
			EU585376	EU585453	EU585530
Y. brevifolia	22	Delmar Road	EU585373	EU585450	EU585527
			EU585374	EU585451	EU585528
Y. brevifolia	23	Sheep Pass	EU585367	EU585444	EU585521
			EU585368	EU585445	EU585522
Y. brevifolia	24	Sawmill Road	EU585369	EU585446	EU585523
			EU585370	EU585447	EU585524
Y. brevifolia	25	Yucca Forest	EU585357	EU585434	EU585511
			EU585358	EU585435	EU585512
Y. brevifolia	26	Dry Lake	EU585351	EU585428	EU585505
			EU585352	EU585429	EU585506
Y. brevifolia	27	Shivwits	EU585377	EU585454	EU585531
			EU585378	EU585455	EU585532
Y. brevifolia	28	Indian Ridge	EU585355	EU585432	EU585509
			EU585356	EU585433	EU585510
Y. brevifolia	29	Kyle Canyon	EU585349	EU585426	EU585503
			EU585350	EU585427	EU585504
Y. brevifolia	30	Lovell Canyon	EU585381	EU585458	EU585535
			EU585382	EU585459	EU585536
Y. brevifolia	31	Jean, NV	EU585365	EU585442	EU585519
			EU585366	EU585443	EU585520
Y. brevifolia	32	Searchlight	EU585359	EU585436	EU585513
			EU585360	EU585437	EU585514
Y. brevifolia	33	Cima	EU585361	EU585438	EU585515
			EU585362	EU585439	EU585516
Y. brevifolia	34	Kingston Mountains	EU585363	EU585440	EU585517
			EU585364	EU585441	EU585518

Taxon	Site #	Site Name	GenBank Accession trnT-L	GenBank Accession trnL-trnF	GenBank Accession clpP intron 2
Y. brevifolia	35	Avawatz Mtns	EU585353	EU585430	EU585507
			EU585354	EU585431	EU585508
Y. brevifolia	36	Dolan Springs	EU585383	EU585460	EU585537
			EU585384	EU585461	EU585538
Y. brevifolia	37	Alamo Crossing	EU585381	EU585458	EU585535
			EU585382	EU585459	EU585536
Y. brevifolia	38	Alamo Lake	EU585385	EU585462	EU585539
			EU585386	EU585463	EU585540
Y. brevifolia	39	Joshua Tree Parkway	EU585379	EU585456	EU585533
			EU585380	EU585457	EU585534
