# Supplementary material for: Comparative Phylogeography of a Coevolved Community: Concerted Population Expansions in Joshua Trees and Four Yucca Moths
Source: PLoS One. 2011 Oct 18;6(10):e25628. doi: 10.1371/journal.pone.0025628 (PMC3196504; doi:10.1371/journal.pone.0025628)
Supplement: Table S5 — Location, carbon-14 ages, and calendar year ages estimated using CALIB 5.0.2 of fossil records for Y. brevifolia.. (PDF) [file pone.0025628.s007.pdf]

Table S5: Location, Carbon-14 ages, and calendar year ages estimated using CALIB 5.0.2 of fossil records for *Y. brevifolia.*

Site Name	Reference	Latitude	Longitude	Elevation (m)	C14 Age	C14 Error	Calendar Years	Calendar Min	Calendar Max
Kofa Mountains	[35]	33.433	-114.1	551	11450	400	13365	12962	13709
SE Sheep Range	[36]	36.467	-115.25	1580	11550	150	13416	13261	13567
Whipple Mountains	[37]	34.217	-114.367	320	11650	190	13515	13324	13693
Western Grand Canyon	[38]	36.1	-113.933	535	12230	350	14282	13777	14769
Waterman Mountains	[39]	32.35	-111.458	795	12530	140	14638	14348	14934
Whipple Mountains	[37]	34.217	-114.367	365	12670	260	14829	14349	15228
Picacho Peak	[40]	32.967	-114.833	285	12730	410	14924	14219	15463
Scodie Mountains	[41]	35.6	-117.95	1130	12820	400	15060	14387	15650
Scodie Mountains	[41]	35.6	-117.95	1130	12960	270	15308	14931	15760
Alabama Hills	[42]	36.625	-118.125	1460	13350	110	15846	15604	16063
Kofa Mountains	[35]	33.433	-114.1	551	13400	250	15902	15513	16268
Whipple Mountains	[37]	34.267	-114.417	520	13810	270	16449	16045	16847
Puerto Blanco Mountains	[43]	31.967	-112.783	565	14120	260	16899	16440	17311
Amargosa Desert	[17]	36.569	-116.086	910	14810	400	17904	17421	18596
Western Grand Canyon	[38]	36.1	-113.933	530	16330	270	19496	19257	19810
Western Sheep Range	[36]	36.633	-115.283	1855	16490	220	19657	19459	19865
Waterman Mountains	[39]	32.35	-111.458	795	17030	380	20187	19614	19654
Ajo Mountains	[44]	32.117	-112.7	975	17830	870	21207	20194	22192
Artillery Mountains	[37]	34.367	-113.617	723	18320	400	21712	21244	22253
Western Sheep Range	[36]	36.638	-115.278	1855	18890	340	22474	22042	22949
Alabama Hills	[42]	36.625	-118.125	1460	19070	190	22628	22346	22849
West Central Sheep Range	[36]	36.704	-115.267	1630	19200	580	22918	22231	23699
Waterman Mountains	[39]	32.35	-111.458	795	19270	340	22981	22488	23400
SE Sheep Range	[36]	36.467	-115.25	1600	19400	300	23112	22631	23495
Panamint Range	[45]	36.583	-117.333	425	19550	650	23333	22467	24102
Alabama Hills	[42]	36.625	-118.125	1460	20310	220	24331	24004	24595
Ajo Mountains	[44]	32.117	-112.7	975	20490	510	24600	24015	25365
Alabama Hills	[42]	36.625	-118.125	1460	21130	480	25268	24977	26000
