# Supplementary material for: Comparative Phylogeography of a Coevolved Community: Concerted Population Expansions in Joshua Trees and Four Yucca Moths
Source: PLoS One. 2011 Oct 18;6(10):e25628. doi: 10.1371/journal.pone.0025628 (PMC3196504; doi:10.1371/journal.pone.0025628)
Supplement: Table S6 — Summary of Lamarc simulations. (PDF) [file pone.0025628.s008.pdf]

Table S6: Summary of Lamarc Simulations.

Simulated Data

Demographic History

Decline	***Y. brevifolia***			***P. sordidus***			***P. weethumpi***			***T. antithetica***			***T. synthetica***		
	Gene	Mean ML Estimate	Mean ML Confidence Interval	Gene	Mean ML Estimate	Mean ML Confidence Interval	Gene	Mean ML Estimate	Mean ML Confidence Interval	Gene	Mean ML Estimate	Mean ML Confidence Interval	Gen e	Mean ML Estimate	Mean ML Confidence Interval
	cpDNA	0.07 +/- 0.18	-1.07, 1.05	COI	142.79 +/- 24.96	-28.34, 313.64	COI	70.60 +/- 60.58	-19.93, 245..96	COI	154.10 +/- 37.30	-29.85, 337.24	COI	57.45 +/- 25.21	-51.50, 151.87
				EF1a	29.59 +/- 23.14	0.63, 98.02	EF1a	293.41 +/- 167.91	-532.14, 1360.67	EF1a	9.11 +/- 4.40	-20.04, 53.78	EF1a	28.13 +/- 7.57	-0.56, 75.18
				Both	47.00 +/- 28.25	7.86, 81.52	Both	46.41 +/- 49.80	-40.99, 137.70	Both	15.87 +/- 11.08	-17.24, 52.09	Both	21.54+/- 6.59	-5.43, 49.56

Constant Size	***Y. brevifolia***			***P. sordidus***			***P. weethumpi***			***T. antithetica***			***T. synthetica***		
	Gene	Mean ML Estimate	Mean ML Confidence Interval	Gene	Mean ML Estimate	Mean ML Confidence Interval	Gene	Mean ML Estimate	Mean ML Confidence Interval	Gene	Mean ML Estimate	Mean ML Confidence Interval	Gen e	Mean ML Estimate	Mean ML Confidence Interval
	cpDNA	0.41 +/- 0.16	-1.05, 1.93	COI	162.37 +/- 32.66	-45.17, 378.72	COI	71.82 +/- 24.85	-44.01, 362.28	COI	152.23 +/- 44.92	-58.45, 363.41	COI	110.56 +/- 42.55	-42.06, 257.08
				EF1a	25.84 +/- 14.87	-7.23, 118.11	EF1a	353.77 +/- 129.12	-591.05, 1704.37	EF1a	16.43 +/- 9.05	-20.82, 91.90	EF1a	24.96 +/- 5.17	-1.45, 65.73
				Both	37.77 +/- 18.89	-2.14, 84.05	Both	75.93 +/- 35.91	-52.60, 200.03	Both	26.54 +/- 17.76	-19.41, 72.70	Both	21.07 +/- 6.56	-5.46, 45.57

	Y. brevifolia			***P. sordidus***			***P. weethumpi***			***T. antithetica***			***T. synthetica***		
	Gene	Mean ML Estimate	Mean ML Confidence Interval	Gene	Mean ML Estimate	Mean ML Confidence Interval	Gene	Mean ML Estimate	Mean ML Confidence Interval	Gene	Mean ML Estimate	Mean ML Confidence Interval	Gen e	Mean ML Estimate	Mean ML Confidence Interval
Slight Growth	cpDNA	0.65 +/- 0.27	-1.37, 2.89	COI	178.04 +/- 51.17	-75.81, 446.45	COI	173.42 +/- 81.67	-26.49, 679.41	COI	163.58 +/- 45.49	-80.68, 412.42	COI	110.67 +/- 34.97	-61.63, 277.17
				EF1a	27.43 +/- 39.28	-16.25 , 142.41	EF1a	918.66 +/- 349.95	-478.54, 3538.23	EF1a	21.59 +/- 13.55	-30.63, 113.26	EF1a	24.58 +/- 7.02	-4.87, 76.91
				Both	43.55 +/- 49.18	-13.12, 96.55	Both	137.78 +/- 59.17	-91.72, 512.41	Both	23.06 +/- 14.62	-27.14, 79.44	Both	22.50 +/- 9.32	-7.86, 76.91

	Y. brevifolia			***P. sordidus***			**P. weethumpi**			**T. antithetica**			**T. synthetica**		
	Gene	Mean ML Estimate	Mean ML Confidence Interval	Gene	Mean ML Estimate	Mean ML Confidence Interval	Gene	Mean ML Estimate	Mean ML Confidence Interval	Gene	Mean ML Estimate	Mean ML Confidence Interval	Gen e	Mean ML Estimate	Mean ML Confidence Interval
Doubling	cpDNA	1.19 +/- 0.40	-2.34, 5.92	COI	270.91 +/- 103.13	-106.26, 709.41	COI	397.00 +/- 225.87	7.11, 1360.46	COI	297.43 +/- 83.89	-109.41, 776.56	COI	204.71 +/- 101.84	-72.34, 505.18
				EF1a	24.26 +/- 17.75	-16.15, 101.59	EF1a	689.93 +/- 294.12	-570.41, 2703.35	EF1a	64.97 +/- 46.00	-32.48, 286.71	EF1a	34.99 +/- 14.97	-17.31, 165.63
				Both	24.23 +/- 21.96	-18.59, 62.24	Both	205.56 +/- 90.12	-91.72, 512.41	Both	90.93 +/- 62.0	-9.20, 204.23	Both	32.90 +/- 16.44	-17.52, 79.57
